# Comprehensive analysis of the prognostic implication and immune infiltration of CISD2 in diffuse large B-cell lymphoma

**DOI:** 10.3389/fimmu.2023.1277695

**Published:** 2023-12-12

**Authors:** ChaoFeng Zhang, Qi Lin, ChunTuan Li, Yang Qiu, JingYu Chen, XiongPeng Zhu

**Affiliations:** ^1^Department of Haematology, Quanzhou First Hospital Affiliated to Fujian Medical University, Quanzhou, China; ^2^Department of Hematology and Rheumatology, The Affiliated Hospital of Putian University, Putian, China; ^3^The School of Basic Medicine, Putian University, Putian, China; ^4^Department of Pharmacy, The Affiliated Hospital of Putian University, Putian, China

**Keywords:** diffuse large B cell lymphoma, CISD2, prognosis, immune infiltration, risk model

## Abstract

**Background:**

Diffuse large B-cell lymphoma (DLBCL) is the most common B-cell lymphoma in adults. CDGSH iron sulfur domain 2 (CISD2) is an iron–sulfur protein and plays a critical role of cell proliferation. The aberrant expression of CISD2 is associated with the progression of multiple cancers. However, its role in DLBCL remains unclear.

**Methods:**

The differential expression of CISD2 was identified via public databases, and quantitative real-time PCR (qRT-PCR) and western blot were used to identifed the expression of CISD2. We estimated the impact of CISD2 on clinical prognosis using the Kaplan-Meier plotter. Meanwhile, the drug sensitivity of CISD2 was assessed using CellMiner database. The 100 CISD2-related genes from STRING obtained and analyzed using the LASSO Cox regression. A CISD2 related signature for risk model (CISD2Risk) was established. The PPI network of CISD2Risk was performed, and functional enrichment was conducted through the DAVID database. The impacts of CISD2Risk on clinical features were analyzed. ESTIMATE, CIBERSORT, and MCP-counter algorithm were used to identify CISD2Risk associated with immune infiltration. Subsequently, Univariate and multivariate Cox regression analysis were applied, and a prognostic nomogram, accompanied by a calibration curve, was constructed to predict 1-, 3-, and 5-years survival probabilities.

**Results:**

CISD2 was upregulated in DLBCL patients comparing with normal controls via public datasets, similarly, CISD2 was highly expressed in DLBCL cell lines. Overexpression of CISD2 was associated with poor prognosis in DLBCL patients based on the GSE31312, the GSE32918, and GSE93984 datasets (P<0.05). Nine drugs was considered as a potential therapeutic agents for CISD2. By using the LASSO cox regression, twenty seven genes were identified to construct CISD2Risk, and biological functions of these genes might be involved in apoptosis and P53 signaling pathway. The high CISD2Risk value had a worse prognosis and therapeutic effect (P<0.05). The higher stromal score, immune score, and ESTIMATE score were associated with lowe CISD2Risk value, CISD2Risk was negatively correlated with several immune infiltrating cells (macrophages M0 and M1, CD8 T cells, CD4 naïve T cells, NK cell, etc) that might be correlated with better prognosis. Additionally, The high CISD2Risk was identified as an independent prognostic factor for DLBCL patients using both univariate and multivariate Cox regression. The nomogram produced accurate predictions and the calibration curves were in good agreement.

**Conclusion:**

Our study demonstrates that high expression of CISD2 in DLBCL patients is associated with poor prognosis. We have successfully constructed and validated a good prognostic prediction and efficacy monitoring for CISD2Risk that included 27 genes. Meanwhile, CISD2Risk may be a promising evaluator for immune infiltration and serve as a reference for clinical decision-making in DLBCL patients.

## Introduction

Diffuse large B-cell lymphoma (DLBCL) is the most common subtype of non-Hodgkin lymphoma (NHL) (1, 2), accounting for approximately 30–40% of NHL cases. DLBCL is a clinically and biologically heterogeneous disease with variable responses to treatment and prognoses (1–3). R-CHOP (rituximab, cyclophosphamide, doxorubicin, vincristine, and prednisone) has become the standard treatment for DLBCL due to its clinical efficacy and well-established safety (3). There are some risk stratifications, such as activated B-cell (ABC) origin, BCL2/MYC double-expression, and high International Prognostic Index (IPI) score (3, 4), that are associated with poor prognosis, aggressive disease behavior, or resistance to R-CHOP in DLBCL patients. Improved understanding of the factors influencing DLBCL prognosis is crucial for refining risk stratification, tailoring treatment approaches, and ultimately enhancing clinical benefit and overall survival (3, 5).

CDGSH iron-sulfur domain-containing protein 2 (CISD2), also known as mitoNEET, is anchored to the mitochondrial outer membrane (MOM) (6, 7). It believes that CISD2 is associated with lifespan and health span (8), and overexpression of CISD2 might restrain age-associated degeneration of the skin, skeletal muscles, neurons, and cardiac system in aging (7, 9). CISD2 is also involved in the development and progression of multiple cancer types, including breast cancer (10), lung cancer (11), and colorectal cancer (12). Upregulation of CISD2 has often been correlated with aggressive tumor characteristics such as increased tumor size and advanced clinical stage (7, 13, 14). In tumorigenesis, CISD2 can regulate cancer cell growth, proliferation, invasion, biosynthesis, and progression through various cellular processes, including mitochondrial iron metabolism, redox regulation, lipid metabolism, and cellular stress response (7, 9). Moreover, inhibition of CISD2 could improve the chemosensitivity of tumors through increasing cell autophagy and ferroptosis (15, 16). However, knowledge about the biological function of CISD2 in DLBCL is meager.

This study aimed to depict the expression profiles of CISD2 and to analyze its prognostic role and immune infiltration in DLBCL through bioinformatics analysis and to clarify its probable mechanisms. We indicated that high CISD2 acted as a biomarker and an indicator of an adverse prognosis among patients with DLBCL. Taken together, these findings provided evidence that CISD2 is important in the occurrence and development of DLBCL and suggested that CISD2 may be a new biomarker and a novel therapeutic target for DLBCL.

## Materials and methods

### Data collection

The public electronic datasets extracted from The Cancer Genome Atlas (TCGA-DLBC) (n = 47), Genotype-Tissue Expression (GTEx) (n = 444), which were downloaded from UCSC Xena (https://xena.ucsc.edu/), and the Gene Expression Omnibus (GEO, https://www.ncbi.nlm.nih.gov/geo/), including GSE83632 (17) (n = 163), GSE31312 (18) (n = 498), GSE32918 (19) (n = 172), GSE93984 (20) (n = 88), GSE117556 (21) (n = 928), and GSE181063 (22) (n = 1311). The general information and clinical metadata were obtained and provided in **Supplementary Table S1**. Three healthy volunteers were recruited in our institution, and peripheral blood mononuclear cell (PBMC) were extracted, this protocol was approved by the ethics committee of Quanzhou First Hospital Affiliated to Fujian Medical University (No. [2023]K096).

### Cell lines culture and expression validation

The lymphoblastoid cell line GM12878 (BeNa, China), DLBCL cell lines DB (Procell, China), SUDHL4 (Meisen, China), and SUDHL2 (A gift from Eatern-Sounth University), were used and cultured in RPMI-1640 (Biosharp, China) supplemented with 10% fetal bovine serum (FBS, Gibco, USA), 1% streptomycin, and penicillin (Gibco, USA). The expression of CISD2 in cell lines was validated through quantitative real-time polymerase chain reaction (qRT-PCR) and western blotting analysis. First, the total RNA was extracted using TRIzol reagent (Invitrogen, US) and reverse-transcribed into complementary DNA (cDNA) for qRT-PCR following the manufacturer’s instructions. The primer sequences of CISD2 and β-actin (As an endogenous control) used in the experiment are illustrated in **Supplementary Table S2**. Second, the protein was collected using RIPA buffer (Beyotime, China) with 1% PMSF (Beyotime, China), and the concentration of protein was measured using a BCA protein assay kit (Beyotime, China). Then, the extracted protein was loaded onto a 12.5% SDS-PAGE gel (Meilunbio, China) and transferred onto Polyvinylidene fluoride (PVDF) membranes. The membranes were incubated with the anti-CISD2 primary antibody (1:1000, Proteintech, China) at 4°C overnight. After the membranes were incubated with goat anti-mouse IgG (1:10000, Beyotime, China) At room temperature, the level of protein was detected using BeyoECL Plus (Beyotime, China) and quantified using Fiji (version 2.9, fiji.sc).

### Expression analysis and survival analysis

The differential expression of CISD2 between DLBCL patients and healthy donors was generated using the TCGA-DLBC, GTEx, and GSE83632 datasets. The protein expression of CISD2 was explored through The Human Protein Altas (HPA, https://www.proteinatlas.org). And the receiver operating characteristic (ROC) curve was plotted for the performance of distinguishing between them. Meanwhile, we attempted to investigate the prognostic role of CISD2 in multiple GEO datasets, including GSE31312, GSE32918, and GSE93984 datasets, using the survival package.

### Drug sensitivity assessment

CellMiner database (www.discover.nci.nih.gov) (23) was used to assess the drug sensitivity analysis, RNA expression data (RNA: RNA-seq) and drug data (Compound activity: DTP NCI-60), which the drugs were selected though approving by clinical trial and FDA, were downloaded. The Pearson correlation coefficient between CISD2 expression and drugs was calculated and screened (|Pearson| > 0.03 and *P*  <  0.01) using impute and limma (24) packages.

### Development of a CISD2-related risk model

The TOP 100 CISD2-related genes were downloaded from the Search Tool for the Retrieval of Interacting Genes/Proteins (STRING, https://version-12-0.string-db.org/, Version 12.0) with at least a medium confidence score (0.400). Based on the GSE117556 dataset, these genes were inputted into the least absolute shrinkage and selection operator (LASSO) Cox regression using the glmnet package (25), a CISD2 related signature for risk stratification (CISD2Risk) was developed and determined, and the risk score was generated: risk score = ∑β_i_x_i_.

### Performance assessment for CISD2Risk

Aiming to elucidate CISD2Risk-related biological function and interaction, we imported genes of CISD2Risk to STRING and performed the protein-protein interaction (PPI) network, in which the association was represented via a confidence score greater than 0.400 and a P-value less than 0.05. Next, we uploaded these genes to the Database for Annotation, Visualization and Integrated Discovery (DAVID) (26), and Gene Ontology (GO) (27) and Kyoto Encyclopedia of Genes and Genome (KEGG) analysis (28) were executed. Based on the GSE117556 as training datasets and the GSE181063 as validation datasets, expression analysis, survival analysis, univariate Cox analysis, and multivariate Cox analysis were adopted to appraise the association of the CISD2Risk and clinical characteristics with OS.

### CISD2Risk associated with immune infiltration

To explore the potential immune infiltration contributing to CISD2Risk, we qualified the tumor microenvironment, including the stroma score, immune score, and estimate score, using the estimate package (29). The cell type identification by estimating relative subsets of RNA transcripts (CIBERSORT) algorithm (30) was used to evaluate 22 types of immune cell infiltration in the GSE117556 and GSE181063 datasets, and the difference between the CISD2Risk value and the abundances of immune cells was estimated. And the Microenvironment Cell Populations-counter (MCP-counter) algorithm (31) that could allow use of the transcriptome data to quantify the absolute abundance of 8 immune cells and 2 stromal cells was analyzed.

### Prognostic implication of CISD2Risk

Meanwhile, we developed the nomograms using the rms package, and the time-dependent receiver operating characteristic (ROC) curves were plotted to determine the prognostic accuracy of the CISD2Risk using the timeROC package (32), and the probability of 1-, 3-, and 5-year OS can be obtained. A calibration curve was used to visualize the deviation of predicted probabilities from what actually happened. The concordance index (C-index) was used to measure the predictive accuracy of the nomogram.

### Statistical analysis

Data are expressed as the mean ± standard deviation (SD). Comparisons between two groups were analyzed using the Student’s t-test (two-tailed). Comparisons among groups were analyzed using a one-Way ANOVA followed by the Tukey test. All analyses were performed with R programming (version 4.2.1). *P* < 0.05 was considered to indicate a statistically significant difference.

## Results

### Upregulated CISD2 expression in DLBCL

**Figure 1:**
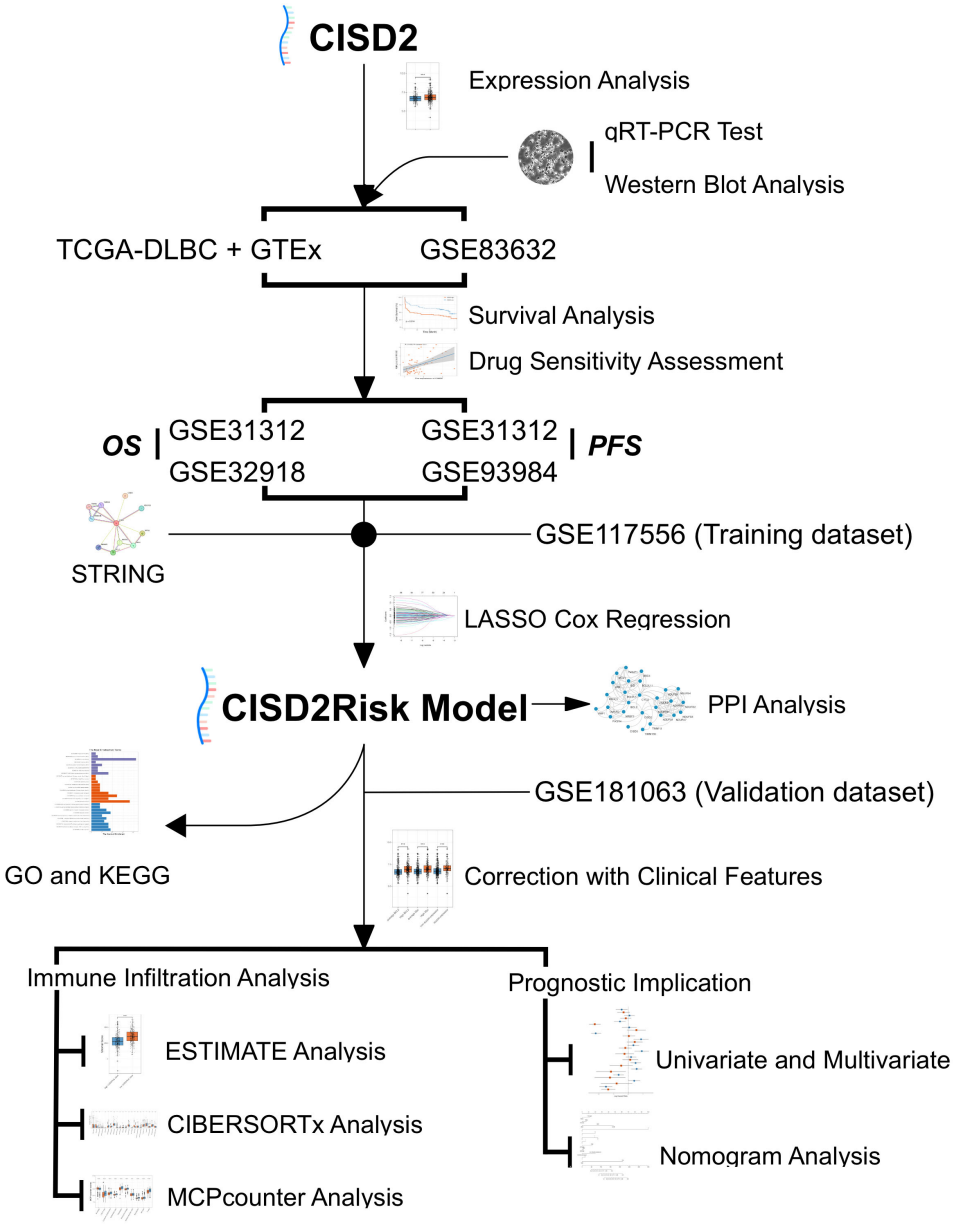
Study flowchart. DLBCL, Diffuse large B-cell lymphoma; GO, Gene Ontology; GSE, Gene Expression Omnibus Series; GTEx, The Genotype-Tissue Expression; KEGG, the Kyoto Encyclopedia of Genes and Genomes; LASSO, the least absolute shrinkage and selection operator regression; MCP-counter, Microenvironment Cell Populations-counter; OS, over survival; PFS, progression-free survival; PPI, Protein-protein interaction; qRT-PCR, real-time reverse transcription-PCR; STRING, the Retrieval of Interacting Genes/Proteins; TCGA, The Cancer Genome Atlas.

**Figure 2:**
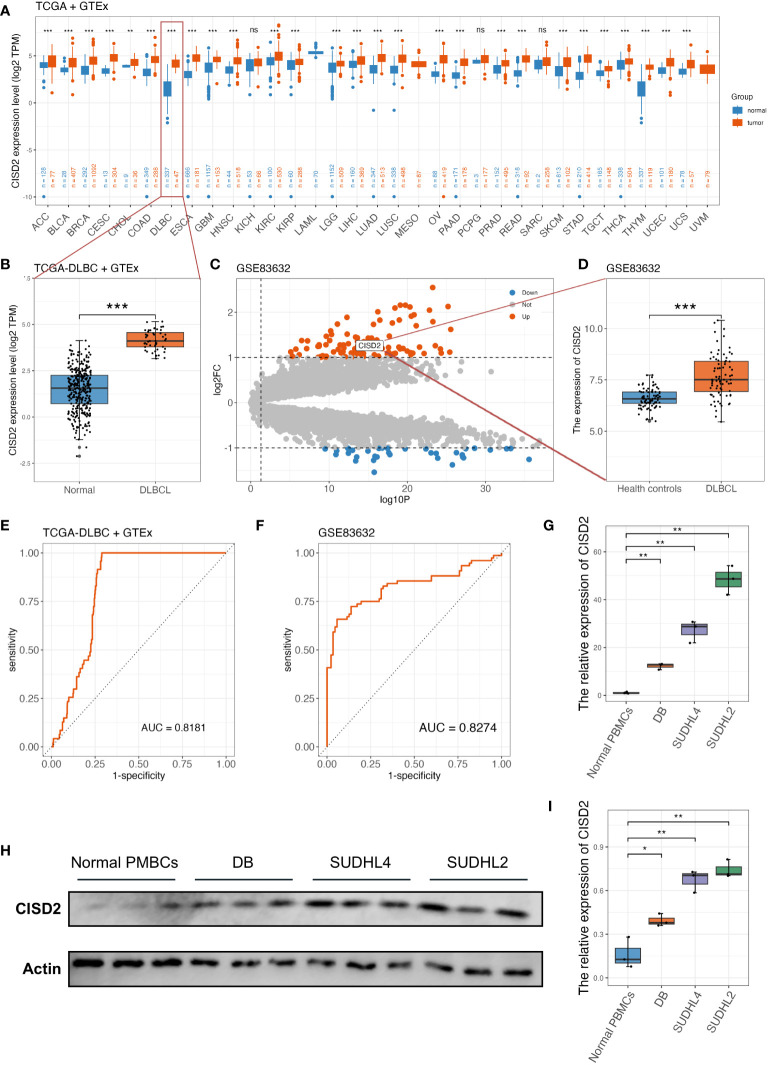
The upregulated expression of CISD2 in DLBCL. **(A)** The gene expression profile of CISD2 in different types of tumors and its homologous normal tissues, data was extracted from TCGA and GTEx. **(B)** CISD2 expression in DLBCL tissue (TCGA-DLBC, n = 47) compared with the whole blood excluded EBV transformed lymphocytes in GTEx cohort (n = 337). **(C)** The volcano plot based on GSE83632 datasets, CISD2 was located in the area of upregulation, adjust *P* value < 0.05 and fold change < 1. **(D)** CISD2 expression in DLBCL whole blood samples (n = 76) compared with healthy controls (n = 87) based on GSE83632 dataset. The ROC curves and AUC for evaluating the prediction accuracy of CISD2 in the network analysis of TCGA-DLBC and GTEx gene expression datasets **(E)**, and GSE83632 dataset **(F)**. The expression of CISD2 in different B lymphocyte cell lines, including normal PBMCs, SUDHL2, SUDHL4, and DB. A qRT-PCR analysis **(G)**, and a WB analysis **(H, I)**. AUC, area under curve; DLBCL, Diffuse large B-cell lymphoma; GSE, Gene Expression Omnibus Series; GTEx, The Genotype-Tissue Expression; PBMC, peripheral blood mononuclear cell; qRT-PCR, real-time reverse transcription-PCR; ROC, receiver operating characteristic; WB, western blotting. *** *P* < 0.001, ** *P* < 0.01, * *P* < 0.05, ns, not signifcance.

**Figure 1** illustrates the workflow in this study. Based on TIMER2.0 (http://timer.cistrome.org), we found that CISD2 expression was upregulated in numerous tumors (7) (**Supplementary Figure S1A**), including lung adenocarcinoma (LUAD) (33), breast cancer (BRCA) (10, 34), and liver cancer (LIHC) (35). Due to the lack of normal control, the whole blood cohort was often used as a reference to TCGA-DLBC. After excluding Epstein Barr virus (EBV) transformed lymphocytes, a total of 337 whole blood specimens were enrolled. In the comparison of gene expression between TCGA tumor and GTEx normal datasets (**Figures 2A**). CISD2 expression was dramatically increased in DLBCL samples in TCGA-DLBC compared with 337 whole blood specimens in GTEx dataset(*P* < 0.05, **Figure 2B**). We also analyzed CISD2 expression patterns in the GSE83632 datasets, which enrolls 76 DLBCL patients and 87 healthy controls (HCs). As shown in **Figures 2C, D**, the result showed CISD2 expression in DLBCL was higher than in HCs (*P* < 0.05). In order to assess the performance of the CISD2 expression for the predictor variable. First, comparing TCGA-DLBC with whole blood samples in GTEx dataset, the area under the curve (AUC) of the ROC curve was 0.818 (95% CI: 0.780-0.856, **Figure 2E**). Meanwhile, the AUC value of 0.8274 (95% CI: 0.759-0.896, **Figure 2F**) was showed in the GSE83632 dataset. On the other hand, using HPA dataset, the protein expression of CISD2 in lymphoma tissues were higher than lymph node tissues (**Supplementary Figure S1B**). For further comparison of CISD2 expression among B-cell lines, first, CISD2 expression of DLBCL cell lines, including DB, SUDHL4, and SUDHL2, was upregulated compared with normal B cell lines (GM12878) through WB analysis (**Supplementary Figures S1C, D**) and qRT-PCR analysis (**Supplementary Figure S1E**). Second, the PBMCs extracted from three healthy volunteers, comparison with DLBCL cell lines, the CISD2 expression in PBMCs downregulated using qRT-PCR and WB analysis(*P* < 0.05, **Figures 2G–I**). These evidences indicated that CISD2 has auxiliary diagnostic significance in distinguishing DLBCL samples from normal samples.

### Prognostic role of CISD2 expression in DLBCL

**Figure 3:**
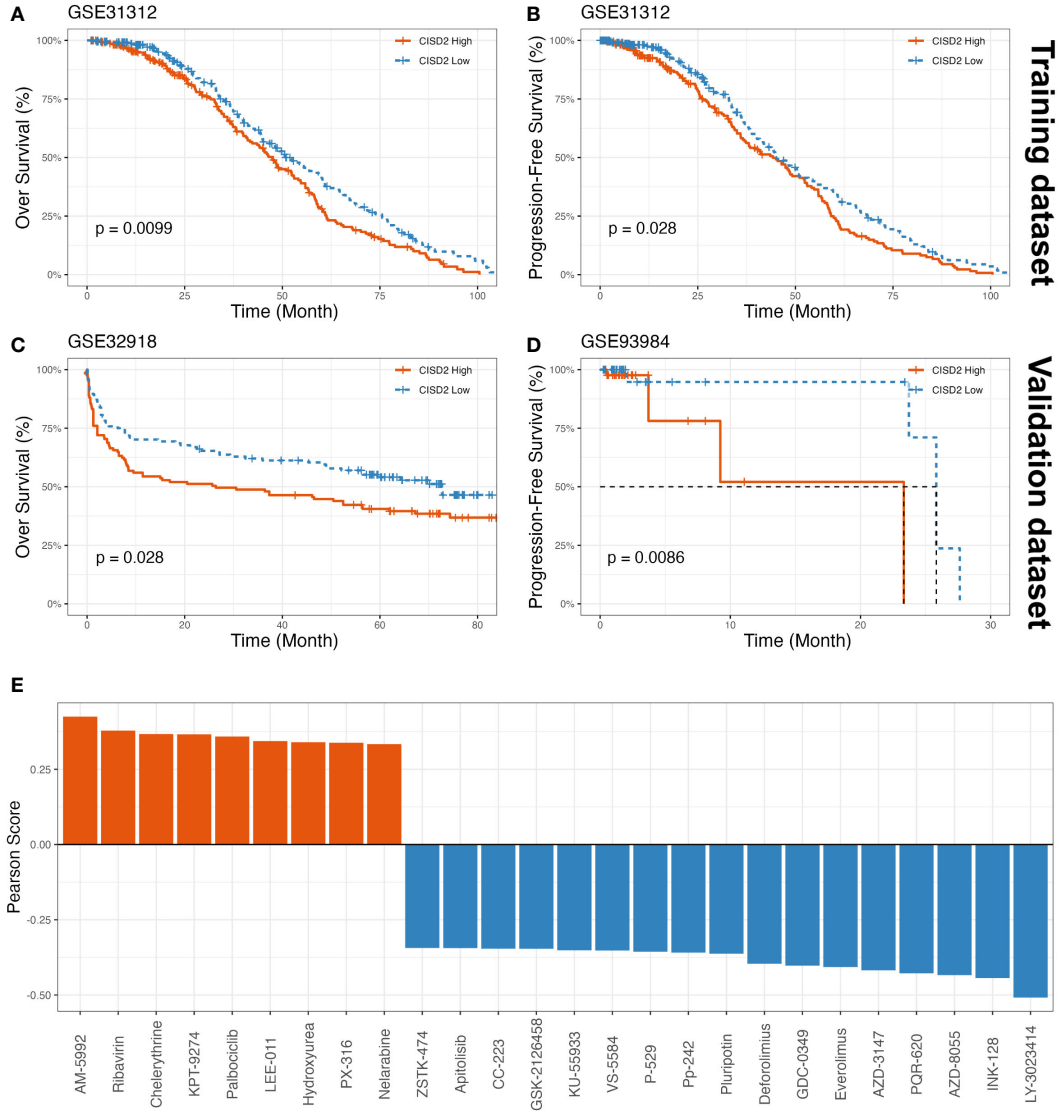
The prognostic value of CISD2 expression in DLBCL and drug sensitivity assessment of CISD2. The KM curves showed OS **(A)** and PFS **(B)** based on GSE31312 as training datasets, on the other part, the KM curves showed OS based on GSE32918 as a validation dataset **(C)**, and PFS based on GSE93984 as a validation dataset **(D)**. **(E)** The drug sensitivity analysis was showed, a total of 9 drugs were positively associated with CISD2 expression, and 17 drugs suggested negative correlation though CellMiner database. GSE, Gene Expression Omnibus Series; KM, Kaplan–Meier; OS, over survival; PFS, progression-free survival.

To determine whether CISD2 could have a novel prognostic value in DLBCL, we analyzed its prognostic significance in DLBCL patients using a Kaplan-Meier (KM) curve based on GEO datasets. As shown in **Figures 3A, C**, upregulated CISD2 expression was associated with poor over survival (OS) in both the GSE31312 dataset (Hazard Ratio (HR) = 0.746, 95% CI: 0.594-0.938, *P* = 0.01) and the GSE32918 dataset (HR = 0.688, 95% CI: 0.492-0.962, *P* = 0.028) by the KM survival curve analysis. Also, DLBCL with high CISD2 expression showed remarkably worse progression-free survival (PFS) than low CISD2 expression in both the GSE31312 dataset (HR = 0.774, 95% CI: 0.614-0.976, *P* = 0.028) and the GSE93984 dataset (HR = 0.297, 95% CI: 0.086-1.029, *P* = 0.009) (**Figures 3B, D**). For each of the above datasets, patients were stratified into two groups using the median CISD2 expression level as a cutoff and were eliminated if OS or PFS were lower than one month. This suggests that CISD2 expression may influence the prognosis of patients with DLBCL.

### Drug sensitivity assessment of CISD2

Using the CellMiner database (23), the results showed that AM-5992, Ribavirin, Chelerythrine, KPT-9274, Palbociclib, LEE-011, Hydroxyurea, PX-316, and Nelarabine were positively correlated with CISD2 expression (**Figure 3E**, **Supplementary Table S3**). Meanwhile, the scatter plots were provided in **Supplementary Figure S2**. These findings indicated that these small molecule compounds may be potential therapeutic agents for CISD2.

### Development of the CISD2Risk

**Figure 4:**
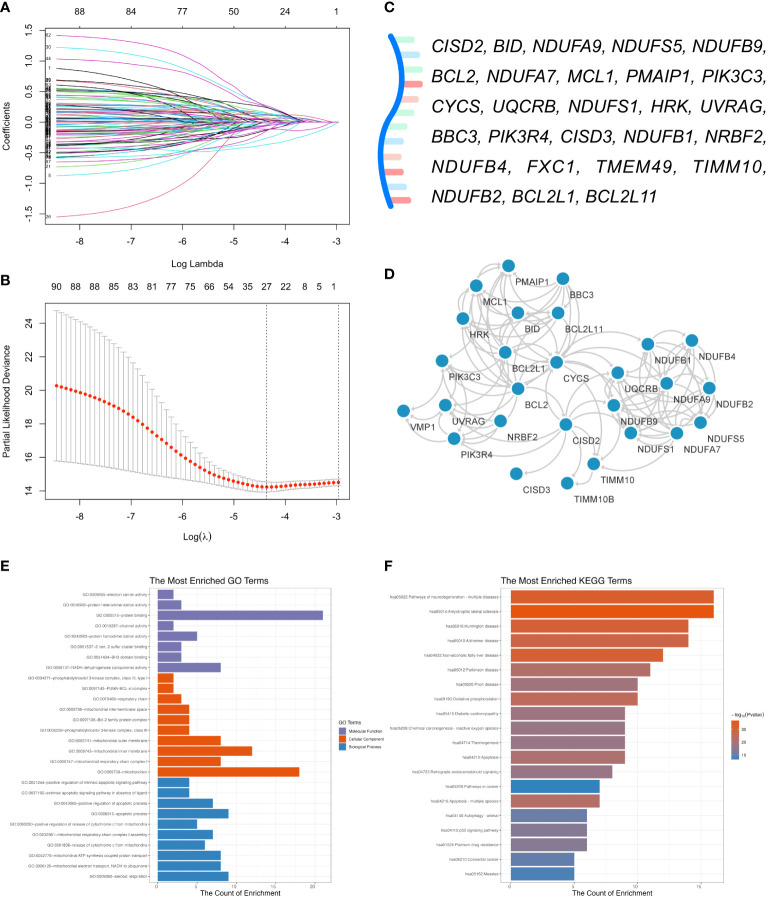
Development of the CISD2 risk model (CISD2Risk). **(A)** The LASSO Cox regression profiles of the CISD2Risk. **(B)** The 27 genes selected using LASSO Cox regression analysis, the two dotted vertical lines were drawn at the optimal scores by lamba.minimum criteria and lamba.1se. **(C)** There were 27 genes enrolled: *CISD2*, *BID*, *NDUFA9*, *NDUFS5*, *NDUFB9*, *BCL2*, *NDUFA7*, *MCL1*, *PMAIP1*, *PIK3C3*, *CYCS*, *UQCRB*, *NDUFS1*, *HRK*, *UVRAG*, *BBC3*, *PIK3R4*, *CISD3*, *NDUFB1*, *NRBF2*, *NDUFB4*, *FXC1*, *TMEM49*, *TIMM10*, *NDUFB2*, *BCL2L1*, and *BCL2L11*. **(D)** The PPI network of 27 genes enrolled in DLBCL patients visualized by the Cytoscape software (version 3.9.1). **(E)** GO enrichment analysis of 27 genes enrolled. **(F)** KEGG pathway analysis of 27 genes enrolled. DLBCL, Diffuse large B-cell lymphoma; GO, Gene Ontology; GSE, Gene Expression Omnibus Series; KEGG, Kyoto Encyclopedia of Genes and Genomes; LASSO, least absolute shrinkage and selection operator; PPI, protein-protein interaction; STRING, Search Tool for the Retrieval of Interacting Genes/Proteins.

To in-depth explore the biological function of CISD2 and its related genes in DLBCL, we collected 100 CISD2-related genes from the STRING website (**Supplementary Figure S3**). A total of 928 patients in the GSE117556 (21) were applied for investigation into their potential effectiveness in this study. After excluding patients with OS times of less than one month, 844 patients were enrolled. The levels of CISD2 related genes were inputted into LASSO Cox regression analysis (**Figures 4A, B**). Twenty-seven genes, including *CISD2*, *BID*, *NDUFA9*, *NDUFS5*, *NDUFB9*, *BCL2*, *NDUFA7*, *MCL1*, *PMAIP1*, *PIK3C3*, *CYCS*, *UQCRB*, *NDUFS1*, *HRK*, *UVRAG*, *BBC3*, *PIK3R4*, *CISD3*, *NDUFB1*, *NRBF2*, *NDUFB4*, *FXC1*, *TMEM49*, *TIMM10*, *NDUFB2*, *BCL2L1*, and *BCL2L11* were evaluated (**Figure 4C**). Therefore, the CISD2Risk was: CISD2Risk = 0.2485 × *CISD2* -0.0610 × *BID* -0.0224 × *NDUFA9 + *0.0176 × *NDUFS5 + *0.2460 × *NDUFB9 + *0.1057 × *BCL2 + *0.04074 × *NDUFA7 + *0.2673 × *MCL1 + *0.1250 × *PMAIP1 + *0.1510 × *PIK3C3 + *0.1428 × *CYCS* + 0.1468 × *UQCRB* + 0.0598 × *NDUFS1 + *0.0390 × *HRK* - 0.2614 × *UVRAG* - 0.0441 × *BBC3 + *0.2380 × *PIK3R4 + *0.1201 × *CISD3* - 0.1192 × *NDUFB1 + *0.0746 × *NRBF2* -0.1411 × *NDUFB4 + *0.0350 × *FXC1* - 0.3655 × *TMEM49* - 0.0525 × *TIMM10* - 0.1292 × *NDUFB2* - 0.1168 × *BCL2L1* - 0.0537 × *BCL2L11*. The PPI network of 27 genes extracted from CISD2Risk was visualized using the Cytoscape software (36) (version 3.9.1, **Figure 4D**).

### Enrichment analysis of CISD2Risk genes in DLBCL

Enrichment analysis of GO enrichment and KEGG pathways was performed based on the CISD2Risk genes. We found enrichment in GO in terms of a few biological processes (BP), such as aerobic respiration, mitochondrial respiratory chain complex I assembly, and apoptotic processes. The main top enrichment cellular component (CC) was the mitochondrion, mitochondrial inner membrane, and mitochondrial outer membrane. And molecular function (MF) enrichment involved protein binding, NADH dehydrogenase (ubiquinone) activity, and BH3 domain binding, as shown in **Figure 4E** and **Supplementary Table S4**. The KEGG pathway analysis showed that oxidative phosphorylation, apoptosis, autophagy, and the P53 signaling pathway are involved (**Figure 4F**, **Supplementary Table S5**).

### Association between CISD2Risk and clinical features in DLBCL

**Figure 5:**
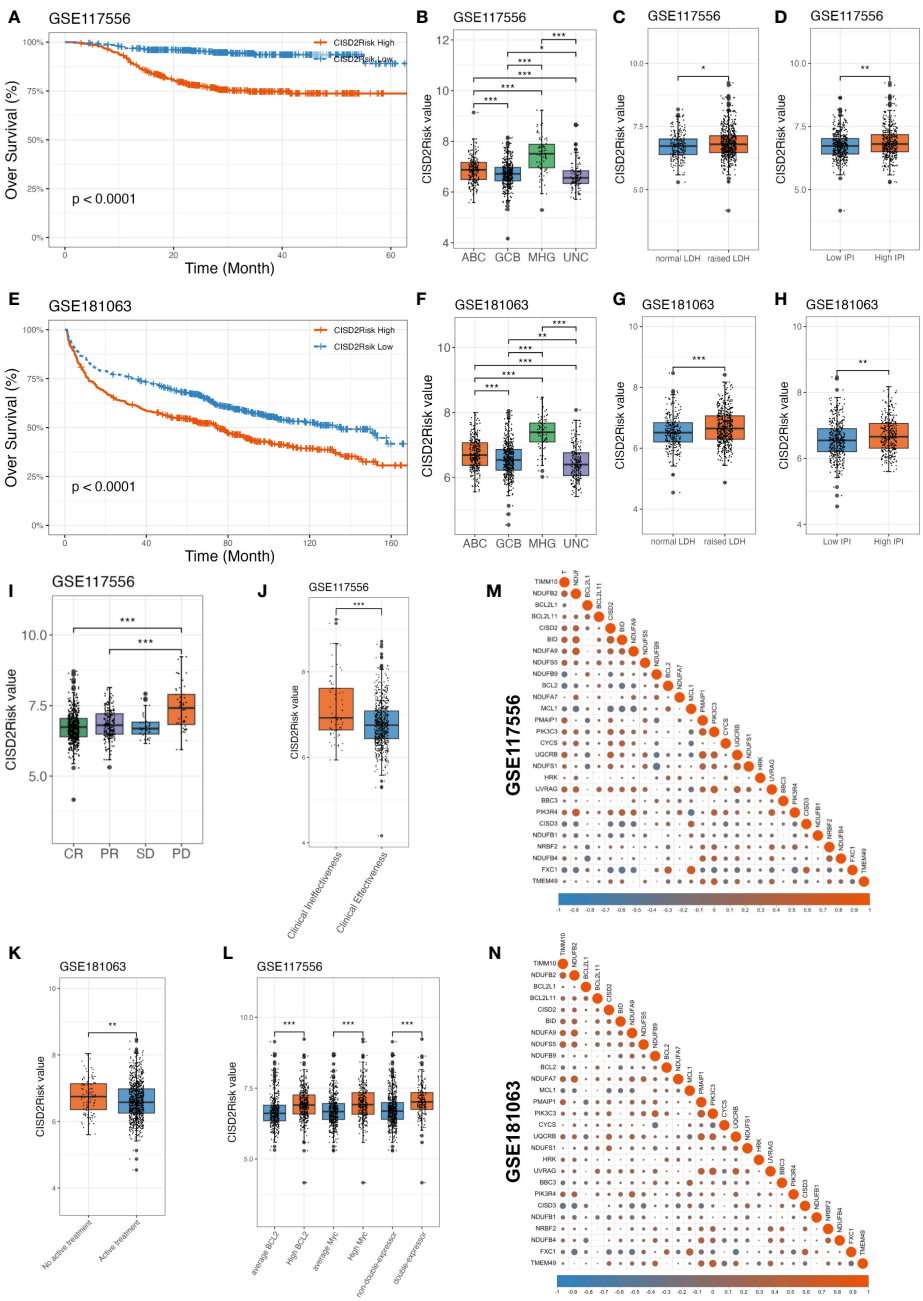
Association between CISD2 expression and clinical features in DLBCL. CISD2Risk was divided into high- and low- groups according to the median based on GSE117556 (training dataset) and GSE181063 (validation dataset). **(A, E)** The KM curves showed that high CISD2Risk group had poor OS. **(B, F)** Comparison among CISD2Risk values of different molecular subgroup. **(C, G)** Comparison between CISD2Risk values of normal and raised LDH. **(D, H)** Comparison between CISD2Risk values of low and high IPI. **(I, J)** Comparison between CISD2Risk values of clinical effectiveness and clinical ineffectiveness based on GSE117556 dataset. **(K)** Comparison between CISD2Risk values of inactive treatment and active treatment DLBCL cases based on GSE181063 dataset. **(L)** Comparison among CISD2Risk values of MYC, BCL2, and double-expressor based on GSE117556 dataset. **(M, N)**. The correlation analysis of 27 genes enrolled by CISD2Risk. CR, complete response; DLBCL, Diffuse large B-cell lymphoma; GSE, Gene Expression Omnibus Series; IPI, international prognostic index; KM, Kaplan–Meier; LDH, lactate dehydrogenase; OS, over survival; PD, progressive disease; PR, partial response; SD, stable disease. *** P < 0.001, ** P < 0.01, * P < 0.05.

Next, we examined the impact of CISD2Risk in DLBCL. Dividing into two groups by the median CISD2Risk value, we found that a high CISD2Risk group was closely related to the relatively poor prognosis of patients with DLBCL in the GSE117556 dataset that enrolled 844 DLBCL patients as a training dataset (*P* < 0.05, **Figure 5A**), and 1058 DLBCL patients in the GSE181063 dataset (Validation dataset) that excluded patients with OS times of less than one month (*P* < 0.05, **Figure 5E**). Sha et al. (21) had defined the molecular high-grade (MHG) subtype of patients with DLBCL that identifies an activated aggressiveness and a poor prognosis (3, 21, 37). In this study, the highest CISD2Risk value in MHG subtype was shown both the GSE117556 and the GSE181063 datasets (*P* < 0.05, **Figures 5B, F**). Generally, the prognosis of DLBCL patients in ABC subtype is inferior to that of the germinal center B cell like (GCB) subtype (38, 39), the CISD2Risk value in ABC subtype DLBCL was higher than GCB subtype DLBCL (*P* < 0.05, **Figures 5B, F**). Similarly, high CISD2Risk patients in ABC, GCB, and MHG DLBCL cases revealed an unfavorable prognosis, compared to low CISD2Risk patients (**Supplementary Figures S4A–C**). We had found that a higher CISD2Risk value in DLBCL patients with raised lactate dehydrogenase (LDH) (greater than 245 U/L) than patients with normal LDH (*P* < 0.05, **Figures 5C, G**). As previously described (40), the IPI score was divided into high and low IPI groups with a value of two as the cut-off, DLBCL patients with high IPI exhibited a significantly higher CISD2Risk value (*P* < 0.05, **Figures 5D, H**), and CISD2Risk valu play a good prognostic role of DLBCL patients both IPI >= 2 and IPI < 2 group (**Supplementary Figures S4D, E**). The clinical effectiveness of treatment for DLBCL is often divided into four categories: complete response (CR), partial response (PR), stable disease (SD), and progressive disease (PD), DLBCL patients achieved PD exhibited higher CISD2Risk value than DLBCL patients achieved CR or PR (*P* < 0.05, **Figure 5I**), we also set patients achieved CR and PR as clinical effectiveness and the others considered as clinical ineffectiveness (41), the result was demonstrated that the DLBCL patients achieved clinical effectiveness manifested lower CISD2Risk value than that achieved clincial ineffectiveness (*P* < 0.05, **Figure 5J**). On the other part, DLBCL cases with low CISD2Risk value were often obtained a curative treatment (*P* < 0.05, **Figures 5K**). Several studies revealed the poor prognosis of MYC and BCL2 and/or BCL6 overexpression in DLBCL, known as double-expressor DLBCL (21, 42). We also reported the overexpression of MYC and BCL2 in DLBCL cases were possiblely associated with CISD2Risk value, and the double-expressor DLBCL exhibited the high CISD2Risk value (*P* < 0.05, **Figure 5L**). These evidences indicated CISD2Risk value was associated with adverse clinical outcomes in DLBCL patients. Subsequencely, we investigated the correlation among included genes in CISD2Risk both the training and validation datasets (**Figures 5M, N**), the results demonstrated that the correlations were similar, indicating that CISD2Risk had relative stability. In addition, CISD2 is related to aging, we divided into two groups based on age, neither greater nor less than 60 years, there was no difference between CISD2 expression and age (**Supplementary Figures S5A–D**). And a high CISD2Risk group was closely related to the relatively poor prognosis of DLBCL patients with different age group (**Supplementary Figures S5E–H**). These results suggested that the prognosis of CISD2Risk was not affected by different ages.

### Relationship between the CISD2Risk and immune infiltration in DLBCL

**Figure 6:**
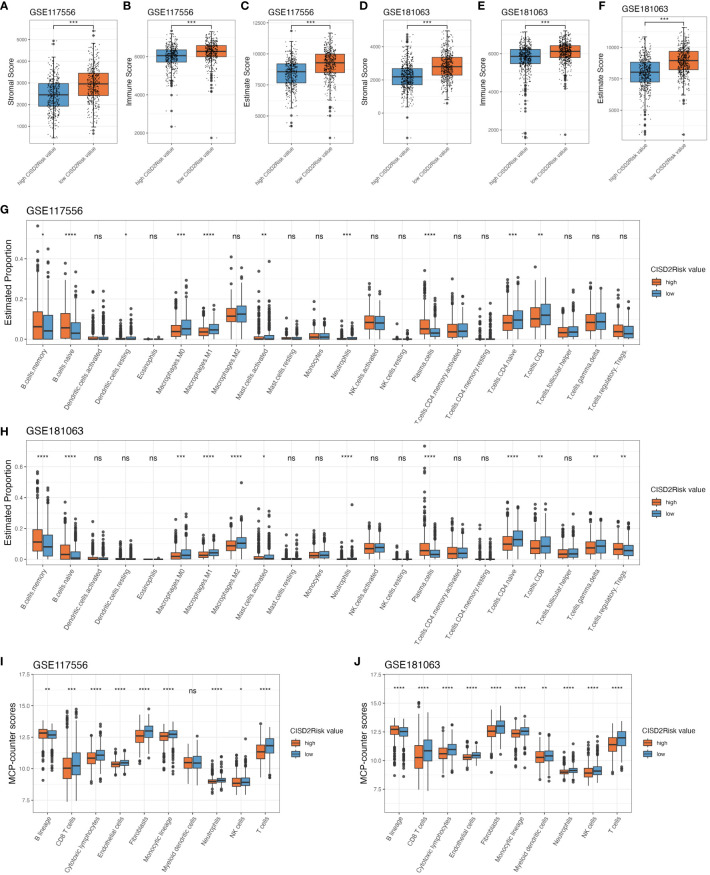
The immune infiltration associations about CISD2Risk in DLBCL based on GSE117556 and GSE1810163 datasets. First, estimate algorithm used to qualitify the tumour microenvironment. **(A, D)** Comparison between the stromal scores of high and low CISD2Risk value, **(B, E)** Comparison between the immune scores of high and low CISD2Risk value, **(C, F)** Comparison between the estimate scores of high and low CISD2Risk value. Second, CIBERSORT algorithm used to evaluate 22 types of immune cell infiltration, **(G, H)** Comparison between the estimate proportion in 22 types of immune cell of high and low CISD2Risk value. Third, MCP-counter algorithm used to analyse 8 types of immune cell, **(I, J)** Comparison between the MCP-counter scores in 8 types of immune cell of high and low CISD2Risk value. CIBERSORT, cell type identification by estimating relative subsets of RNA transcripts; DLBCL, Diffuse large B-cell lymphoma; MCP-counter, Microenvironment Cell Populations-counter. **** P < 0.0001, *** P < 0.001, ** P < 0.01, * P < 0.05, ns, not signifcance.

The tumor immune microenvironment significantly affects the therapeutic effect and prognosis of multiple tumor (43, 44). We introduced the ESTIMATE algorithm to infer the fraction of stromal and immune cells in tumour samples based on single sample gene set enrichment analysis (ssGSEA). A higher stromal (*P* < 0.05, **Figures 6A, D**) or immune scores (*P* < 0.05, **Figures 6B, E**) suggested greater density of stromal or immune cells in the tumour immune microenvironment of DLBCL patients with low CISD2Risk value the ESTIMATE scores that represent the sum of the stromal or immune scores, which can infer tumour purity associated with poor prognosis (29, 45), were negatively correlated with CISD2Risk value (46, 47) (*P* < 0.05, **Figures 6C, F**). The CIBERSORT algorithm (30) was used to estimate the distribution and proportion of 22 immune cell types in DLBCL. The gene expression profiles of the GSE117556 and GSE181063 datasets were inputted into CIBERSORTx (http://cibersortx.stanford.edu). As demonstrated in **Figures 6G, H**, both the GSE117556 and GSE181063 datasets, there were a represented abundance of CD8 T cells, CD4 naïve T cells, macrophages M0, macrophages M1, neutrophils, and activated mast cells had significantly negative correlations with CISD2Risk values (*P* < 0.05). On the contrary, three cells that include naïve B cells, memory B cells, and plasma cells had significantly positive correlations with CISD2Risk value (*P* < 0.05). MCP-counter algorithm (31) aims to estimate immune infiltration by fibroblasts, endothelial cells, and eight immune cells using transcriptomic data. We found that the high CISD2Risk value was associated with significantly decreased abundances of six immune cells, inclinding CD8 T cells, T cells, Natural killer (NK) cells, cytotoxic lymphocytes, neutrophils, and monocytic lineage, while fibroblasts and endothelial cells showed the similar trend both the training and validation datasets. On the other hand, compared with the low CISD2Risk value, the high CISD2Risk exhibited increased proportion of B lineage, as shown in **Figures 6I, J**.

### Prognostic implication of CISD2Risk in DLBCL

**Figure 7:**
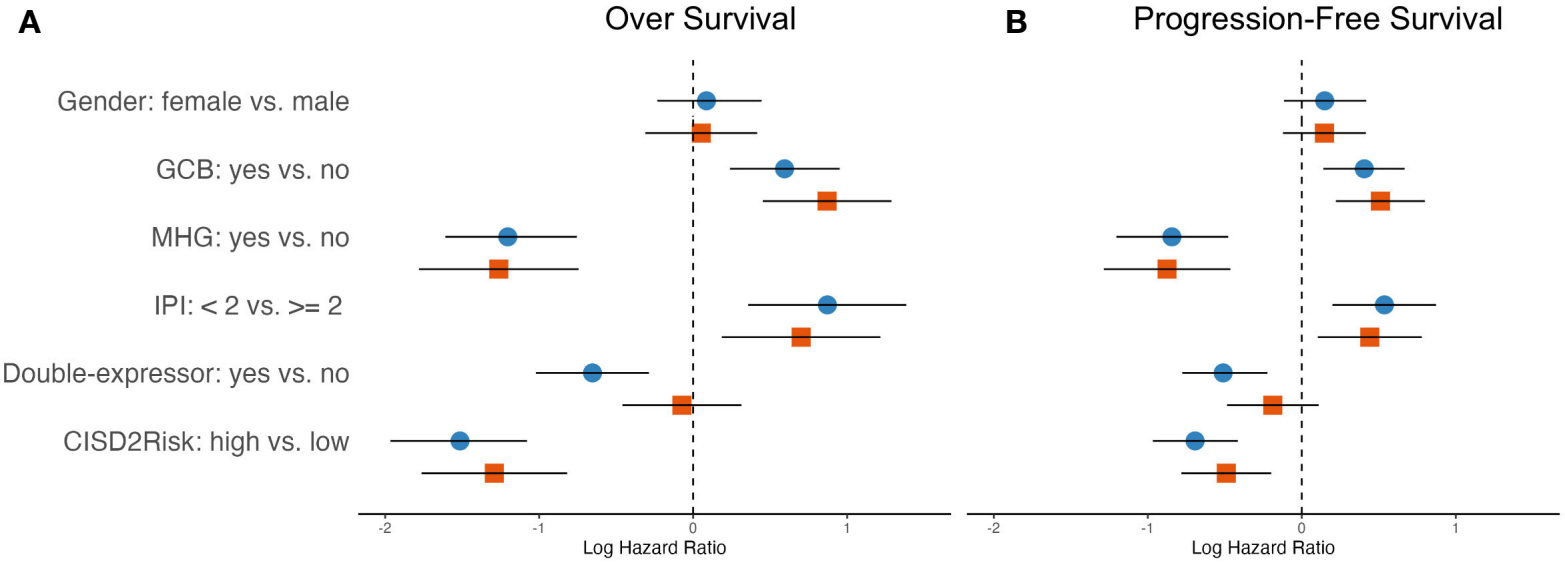
The hazard ratios of clinical features integrated into the OS and PFS showed in the forest plots in DLBCL using univariate and multivariate cox regression analysis based on the training dataset, **(A)** left, OS; **(B)** right, PFS; blue and circle, univariate Cox regression analysis; red and square, multivariate Cox regression analysis. OS, over survival; PFS, progression-free survival.

IPI scoring is a widely used tool to assess the prognosis and predict outcomes for patients with DLBLC, the factors considered in the IPI include ages, clinical stage, elevated serum LDH, Eastern Cooperative Oncology Group Performance Status (ECOG PS), and extranodal sites of disease. To investigate the prognostic values of CISD2Risk in DLBCL, we employed these variables included gender, cell of original (COO), molecular subgroup (21, 22), IPI, double-expressor and status of CISD2Risk divided into high and low levels by median of CISD2Risk value, into univariate and multivariate Cox regression analysis. As shown in **Figure 7**, the results showed that the status of CISD2Risk could be an independent prognostic factor for OS both the GSE117556 (**Figure 7A**) and the GSE181063 (**Supplementary Figure S6**). A forrest plot exploring multiple clinical features for PFS in the GSE117556 dataset was provided, CISD2Risk was also an independent prognostic indicator for PFS (**Figure 7B**).

### Construction and validation of the nomogram in DLBCL

**Figure 8:**
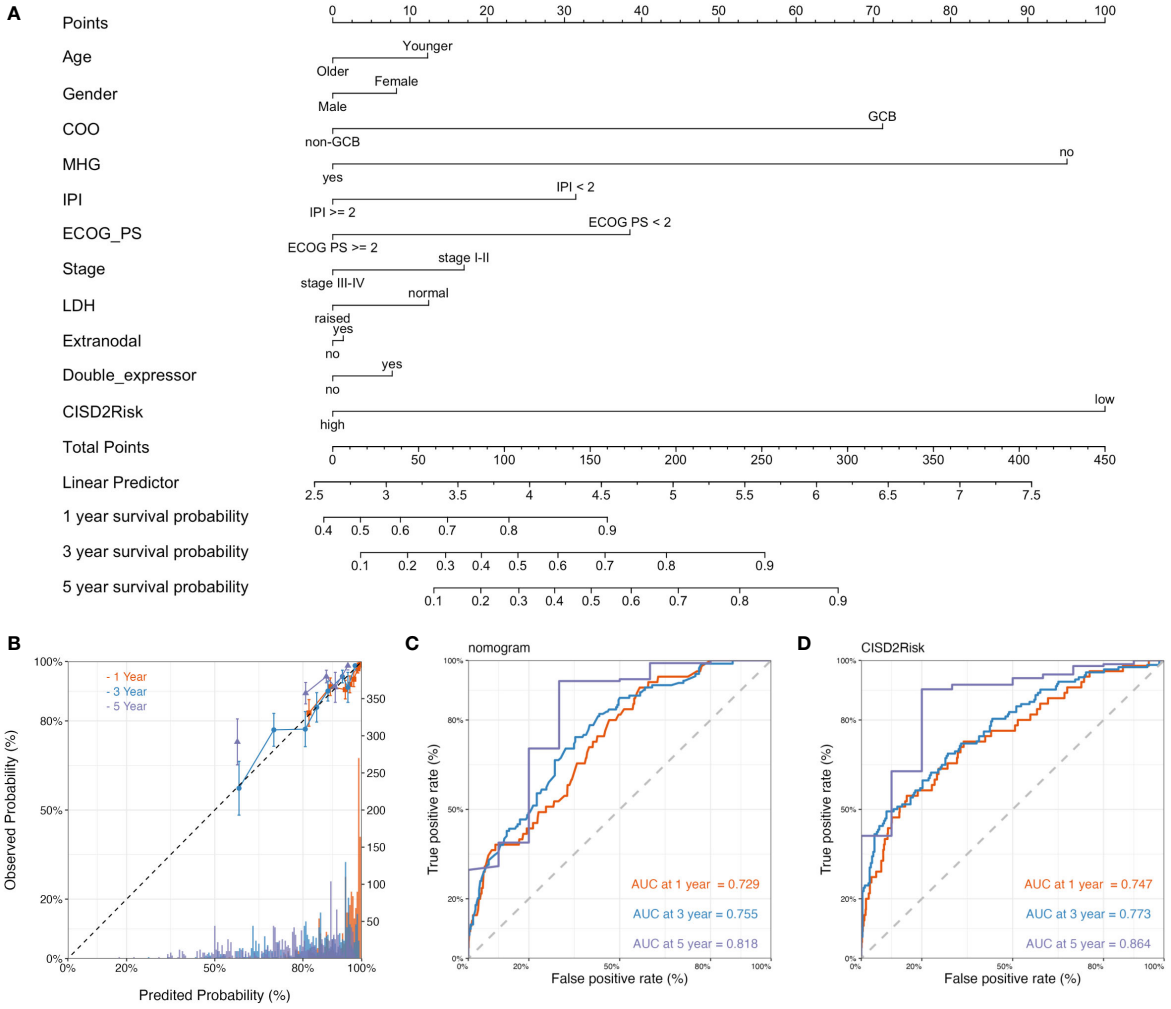
The construction and validation of the nomogram. **(A)** The nomogram plot of the GSE117556 dataset showed the prediction of clinical features including age, gender, COO, molecular subgroup, IPI, ECOG PS, clinical stage, LDH, extranodal, double-expressor, CISD2Risk, and 1-year, 3-year, and 5-year survival probability. **(B)** The calibration curve of 1-year, 3-years, and 5-year survival probability of DLBCL patients, The dashed line represented a perfect uniformity between predicted probability and observed probability. The time-dependent ROC curves for nomogram **(C)** and CISD2Risk **(D)** at 1-year, 3-year, and 5-year for DLBCL, respectively. COO, cell of original; DLBCL, Diffuse large B-cell lymphoma; ECOG PS, Eastern Cooperative Oncology Group performance status; GSE, Gene Expression Omnibus Series; IPI, international prognostic index; LDH, lactate dehydrogenase; ROC, receiver operating characteristic.

We built a prognostic nomogram in DLBCL to anticipate the 1-, 3-, and 5-years OS based on prognostic factors such as age, gender, COO, molecular subgroup (21, 22), IPI, ECOG PS, clinical stage, LDH, extranodal, double-expressor and status of CISD2Risk in the GSE117556 dataset (**Figure 8A**), that the higher total points in the nomogram indicated worse survival. And the C-index of the nomogram was 0.746 (95% CI: 0.743-0.749). While a survival prediction nomogram in the validation dataset was constructed (**Supplementary Figure S7A**) and C-index was 0.732 (95% CI: 0.730-0.734). The calibration curves (The training dataset showed at **Figure 8B**, The valication dataset showed at **Supplementary Figure S4B**) were visualized and indicated acceptable agreement between the predicted survival rate and the actual survival rate, suggesting that these nomograms we constructed might favorably predict the prognosis of patients with DLBCL. The AUC of time-dependent ROC curves (**Figures 8C, D**, and **Supplementary Figures S4C–E**) were presented in **Supplementary Table S6**. These data suggested that the prognostic nomogram constructed by these clinical features and CISD2Risk had a good prediction ability on the prognosis of DLBCL patients.

## Discussion

At present, emerging evidence demonstrates that the pathogenesis of DLBCL is complicated and consistent with aberrant gene expression that affects cell growth (48), invasiveness (49), angiogenesis (50), and apoptosis (51). It is reasonable for us to believe that CISD2 may play a significant role in DLBCL. Using public databases (17–22), we found upregulated CISD2 as an appropriate diagnostic factors and a unfavorable prognostic indicator in DLBCL. Recent studies demonstrated that a clinical risk model included multiple genes is helpful to better implement the eligible diagnostic and the favorably prognostic criteria in DLBCL patients (52–55). Here, we developed a CISD2-related risk model (CISD2Risk) based on CISD2 related genes using LASSO Cox regression analysis in the GSE117556 dataset, and performed external validation (GSE181063 dataset) for its performance. Our results showed that CISD2Risk revealed a good ability to predict survival, and was an independent prognostic factor of DLBCL patients.

There are eight genes (BUB1B, CISD2, KLOTHO, PAWR, PPARG, PTEN, SIRT1, and SIRT6) listed as pro-longevity genes in mammals by the Human Aging Genomic Resources (HAGR) (56). Several studies have showed that some pro-longevity genes (such as PTEN, SIRT1, and SIRT6) influenced the occurrence and development, the drug resistance of DLBCL (57–59). However, the biological function of CISD2 in DLBCL is still unclear. Knockout of CISD2 in mice could cause a number of age-related phenotypes in multiple organs and lead to premature aging (6, 7), suggesting that CISD2 might play a critical role in controlling lifespan. Mechanically, CISD2 could regulate Ca^2+^ homeostasis and maintain mitochondrial function (8, 60). Currently, the role of CISD2 in cancers causes more interest. Sun et al. (13) showed that CISD2 expression was negatively correlated with the survival of patients with glioma, and inhibition of CISD2 might activate BECN-1-mediated autophagy to reduce the proliferation of glioma cells. Cervical cancer patients with higher CISD2 expression had shorter OS and were associated with pelvic lymph node metastasis (61). Upregulation of CISD2 in lung adenocarcinoma (ADC) specimens compared with their adjacent normal counterparts was found (33), and was associated with increased antioxidant capacity in response to elevated ROS levels during the formation and progression of lung cancer (33). In this study, we also found that CISD2 was upregulated in DLBCL compared with NCs, and CISD2 expression was negatively associated with survival, indicating that CISD2 may be involved in the pathologic progression of DLBCL. Numerous studies (6–9) indicated that CISD2 regulates age-associated disorders. The expression of CISD2 could be activated at a late-life stage of aged mice pharmaceutically, hesperetin considered as CISD2 activator enhanced CISD2 expression in order to slow down aging and promote longevity (9). It highlights the urgent need to explore the potential therapeutic strategy for cancer and age-associated diseases based on CISD2 manner. In this study, different age in DLBCL did not affect the CISD2 expression (*P* > 0.05), suggesting that CISD2 might play a role in promoting the development of DLBCL. We also assessed the drug sensitivity, AM-5992, Ribavirin, Chelerythrine, KPT-9274, Palbociclib, LEE-011, Hydroxyurea, PX-316, and Nelarabine were potential therapeutic role for DLBCL.

CISD2 is localized on MOM, ER, and mitochondrial-associated ER membrane (MAM) (7, 9), which is closely related to its biological functions. Natasha et al. indicated that CISD2 could be a physical interaction between BCL2 and BECN1 to antagonize autophagy in response to nutrient stress; the BCL2-CISD2 complex is a requirement for BCL2-mediated depression of ER Ca^2+^ stores (62). It was suggested that CISD2 might be involved in multiple biological processes as an interaction or intermediate. For further investigation of the biological function of CISD2 in DLBCL, we collected 100 CISD2-related genes from STRING and identified 27 genes using LASSO Cox regression analysis. CISD2Risk was developed based on these genes using the GSE117556 datasets. GO and KEGG enrichment analysis of 27 genes revealed that CISD2Risk might be likely to be involved in apoptosis and the P53 signaling pathway and localize on the mitochondrial inner membrane and outer membrane, suggesting that it may participate in mitochondrial apoptosis. Several studies showed that apoptosis proteins such as P53 (63, 64), BCL2 (65–67), and MCL1 (68), directly and indirectly involved in the intrinsic or extrinsic apoptotic pathways in the regulation of pathophysiology and chemotherapy resistance in DLBCL (5, 51, 67, 69).

The efficacy of CISD2Risk was verified from multiple clinical aspects, CISD2Risk showed a good performance associated with clinical factors stratification. MHG is supposed to be an aggressive B-cell lymphoma and show an inferior response to RCHOP treatment (3, 21, 37). In this study, the MHG DLBCL patients exhibited highest CISD2Risk value which indicated a poor prognosis. MHG DLBCL has distinct molecular features with concurrent activation of MYC and BCL2 (21, 42). DLBCL patients with double-expressor that defined by the coexpression of MYC and BCL-2 have a poor prognosis after standard chemoimmunotherapy (21, 42). DLBCL patients with double-expressor has a higher CISD2Risk value than that with non-double-expressor in this study. Also, DLBCL patients with high CISD2Risk value was associated with raised LDH or high IPI level, which might be considered as a predictor of clinical outcomes traditionally (3, 21, 70). Meanwhile, DLBCL patients who were responsed to clinical treatment showed a relatively lower CISD2Risk value. These evidences revealed that a high CISD2Risk value might lead to poor clinical outcomes.

The tumor microenvironment has been considered an important biological aspect of development and occurrence in DLBCL (71–73), which includes multiple immunemodulating mechanisms (73). The stromal cells are well known to be recruited by tumor cells and regulate tumor development, and the immune cells respond to tumor cells by causing inflammatory responses; all of them are involved in the development and occurrence of tumors through immunoregulatory mechanisms (73, 74). We explored the stromal and immune cells in DLBCL using the ESTIMATE algorithm. The high CISD2Risk values were negatively associated with stromal scores, immune scores, and ESTIMATE scores between the training and validation datasets, suggesting poor prognosis and high tumor purity in DLBCL.

Hence, understanding the types and roles of immune cells related to CISD2Risk is crucial to targeting and improving the precise treatment of DLBCL, the CIBERSORT algorithm (30) can be used to accurately estimate the immune composition of the 22 closely related types of immune cells. We found that CISD2Risk value were inversely associated with the infiltration levels of activated mast cells, neutrophils, CD8 T cells, CD4 naïve T cells, macrophages M0, and M1, as well as a positively associated with a high proportion of B cells in DLBCL. Traditionally, Macrophages that acting as sentinels of the tumor microenvironment are extensively involved in the regulation of immune response and homeostasis (75, 76). Macrophages M0 can be polarized into either M1 or M2, activated M1 macrophages produces various pro-inflammatory cytokines to cause tumor damage (76), while M2 decrease inflammation and encourage tissue repair. A high numbers M0, M1 macrophages correlated with better survival in DLBCL (75, 77), and these data are consistent with our data. A lot of studies reported the lower amount of CD4 T cells and CD8 T cells in the lymphoma microenvironment correlated with poor survival (73, 77–79). CD4 naïve T cell is considered essential to guarantee immune competence throughout life, can be activated after interaction with antigen Major Histocompatibility Complex (MHC) and differentiated into memory, effector, and suppressor cells (71, 73). It believed that activated CD4 memory T cell was associated with better survival and overcame some of the chemotherapy resistance (80, 81). CD8 T cells as a key players might have defective cytotoxicity in the process of targeting cancer cells (73, 79). CD8 T cells infiltrating DLBCL that been correlated with better prognosis are highly activated and lack an exhausted phenotype (82). B cells as immunomodulatory cells, positive mediators, and antigen-presenting cells play a role in modulating the immune response to cancer (83). We found that DLBCL with high CISD2Risk value exhibited increased B cell infiltrations in accord with several studies (47, 77, 84, 85). B cells are part of the adaptive immune system and can produce antibodies against cancer cells. There are some studies demonstrated a robust B cell response may indicate an active immune reaction against the tumor. The increased B cells within tumour microenvironment (TME) may reflect an attempt by the immune system to mount an anti-tumor response. On the other hand, inflammatory signals which may be activated by TME of DLBCL can attract immune cells, including B cells, and these immune cells may participate in a proinflammatory response, which can sometimes promote tumor growth and aggressiveness (71, 81, 86). It had been reported that DLBCL recruited T cells and monocytes via CCL5 to support B cells survival and proliferation (87). According to COO, DLBCL was pathologically divided into ABC, GCB, and unclassifiable (UNC) subtypes, the aberrant memory B cells (MBs) might be the true COO for ABC subtype DLBCL (88). The correlation between CISD2Risk and activated B cells may be explained by the pathological features of DLBCL (47, 88). Next, the MCP-counter algorithm (31) can be used to analyze gene expression profiles to estimate the expression levels of multiple tumor-infiltrating lymphocytes. The high CISD2Risk was associated with significantly decreased abundances of NK cells. NK cells recognize and kill cancer cells via releasing cytolytic granules. When DLBCL patients were treated with RCHOP, low amount of NK cell count was associated with shorter PFS and decreased OS compared to patients with high amount of NK cells (89, 90), these data are consistent with our data. These findings suggested CISD2Risk might be used to estimate the anti-tumor immunity of DLBCL patients, but the immune regulation of CISD2Risk needs further investigation.

Additionally, CISD2Risk has effectively and independently determined the prognosis of patients with DLBCL through univariate and multivariate Cox regression. Hence, a novel nomogram was developed that exhibited superior discrimination ability for the prediction of prognosis in DLBCL patients and could be used to guide routine OS for DLBCL patients. Time-dependent ROC curve analysis of the CISD2Risk value revealed a relatively accurate ability to predict OS. Recently, several studies showed some clinical prediction model for DLBCL. A ferroptosis-based risk scoring model of 16 genes for patients with DLBCL was constructed and had good efficacy in predicting survival compared to clinical characteristics (52), similar to Chen et al.’s study (91). Likewise, an immune score model including 22 genes could predict the survival of DLBCL patients and be more accurate than the IPI and Revised International Prognostic Index (R-IPI) (77). 15 differentially expressed genes (DEGs) among metabolic subtypes were used to build a predictive model that could evaluate survival and drug sensitivity in DLBCL patients (78). Different from other risk models (52, 77, 78, 91), we built a risk model based on CISD2 and its related genes, which also has excellent prediction ability in line with those.

Some limitations existed in this study. First, the biological function of CISD2 need to be explored using *in vitro* and *in vivo* experiments. Specifically, the practical effect of drugs that selected should be assessed. Second, both the construction and validation of CISD2Risk were based on retrospective public data; the reliability and applicability of CISD2Risk need to be verified by some clinical experiments. Third, GO and KEGG enrichment analysis of 27 genes revealed that CISD2Risk might be involved in apoptosis, the P53 signaling pathway, and so on; however, the underlying mechanism of these genes needs to be explored in the future.

## Conclusion

In conclusion, our study indicated that upregulated CISD2 was correlated with a poor prognosis. Meanwhile, we developed a CISD2Risk for DLBCL patients that was validated in an independent dataset. CISD2Risk showed better ability of clinical prediction to prognosis. Additionally, CISD2Risk had a capacity for estimation for anti-tumor immunity in DLBCL, suggesting CISD2Risk could be a predictor for clinical prognosis as well as a clinical evaluator for immunotherapy.

## Data availability statement

The datasets presented in this study can be found in online repositories. The names of the repository/repositories and accession number(s) can be found in the article/**Supplementary Material**.

## Ethics statement

The studies involving human participants were reviewed and approved by the ethics committee of Quanzhou First Hospital Affiliated to Fujian Medical University (No. [2023]K096).

## Author contributions

CZ: Funding acquisition, Methodology, Visualization, Writing – original draft. QL: Data curation, Investigation, Visualization, Writing – original draft. CL: Writing – review & editing. YQ: Investigation, Writing – original draft. JC: Investigation, Writing – original draft. XZ: Writing – review & editing.
