# Supplementary material for: Comprehensive analysis of the prognostic implication and immune infiltration of CISD2 in diffuse large B-cell lymphoma
Source: Front Immunol. 2023 Dec 12;14:1277695. doi: 10.3389/fimmu.2023.1277695 (PMC10754510; doi:10.3389/fimmu.2023.1277695)
Supplement: Supplementary file 1 [file DataSheet_1.docx]

Supplementary Material

# Supplementary Data

We have provided the available data of study in the manuscript and supplementary file. The R script is in https://www.github.com at [https://github.com/linqitc/MyRScript/blob/master/CISD2.r.](https://raw.githubusercontent.com/linqitc/MyRScript/master/Meta-analysis_LncRNA.r.) It included our analysis process of study using R programming mainly.

# Supplementary Figures and Tables

## Supplementary Figures


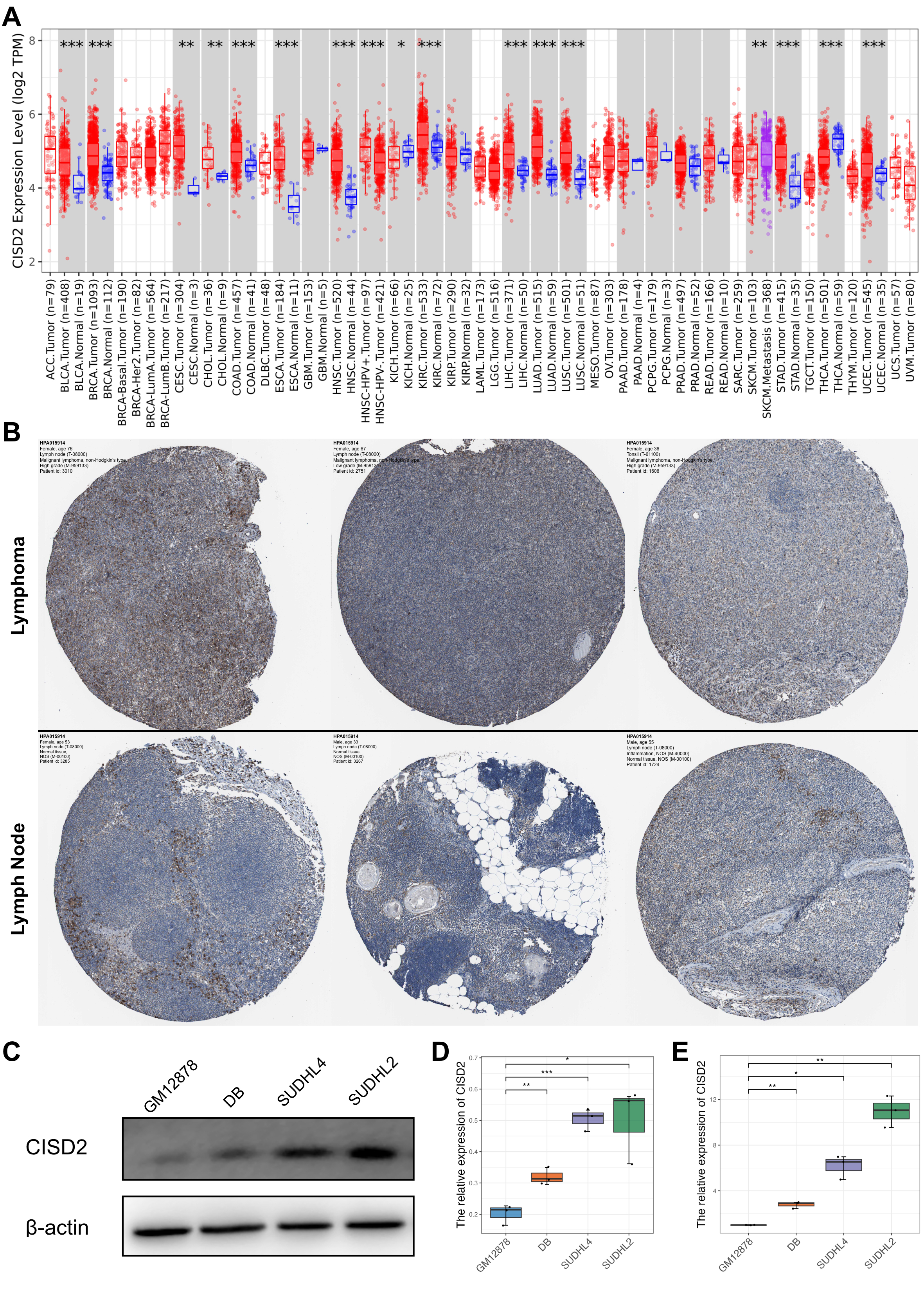


**Supplementary Figure S1.** The upregulated expression of CISD2 in Lymphoma. **A.** The gene expression profile of CISD2 in different types of tumors and its homologous normal tissues using TCGA data through TIMER2.0 (http://timer.cistrome.org). **B.** CISD2 expression in lymphoma tissue and lymph node through The Human Protein Altas (HPA, https://www.proteinatlas.org). The expression of CISD2 in different B lymphocyte cell lines, including GM12878, SUDHL2, SUDHL4, and DB. A WB analysis (**C, D**) and a qRT-PCR analysis (**E**).DLBCL, Diffuse large B-cell lymphoma; GSE, Gene Expression Omnibus Series; GTEx, The Genotype-Tissue Expression; qRT-PCR, real-time reverse transcription-PCR; WB, western blotting. *** *P* < 0.001, ** *P* < 0.01, * *P* < 0.05, ns, not signifcance.


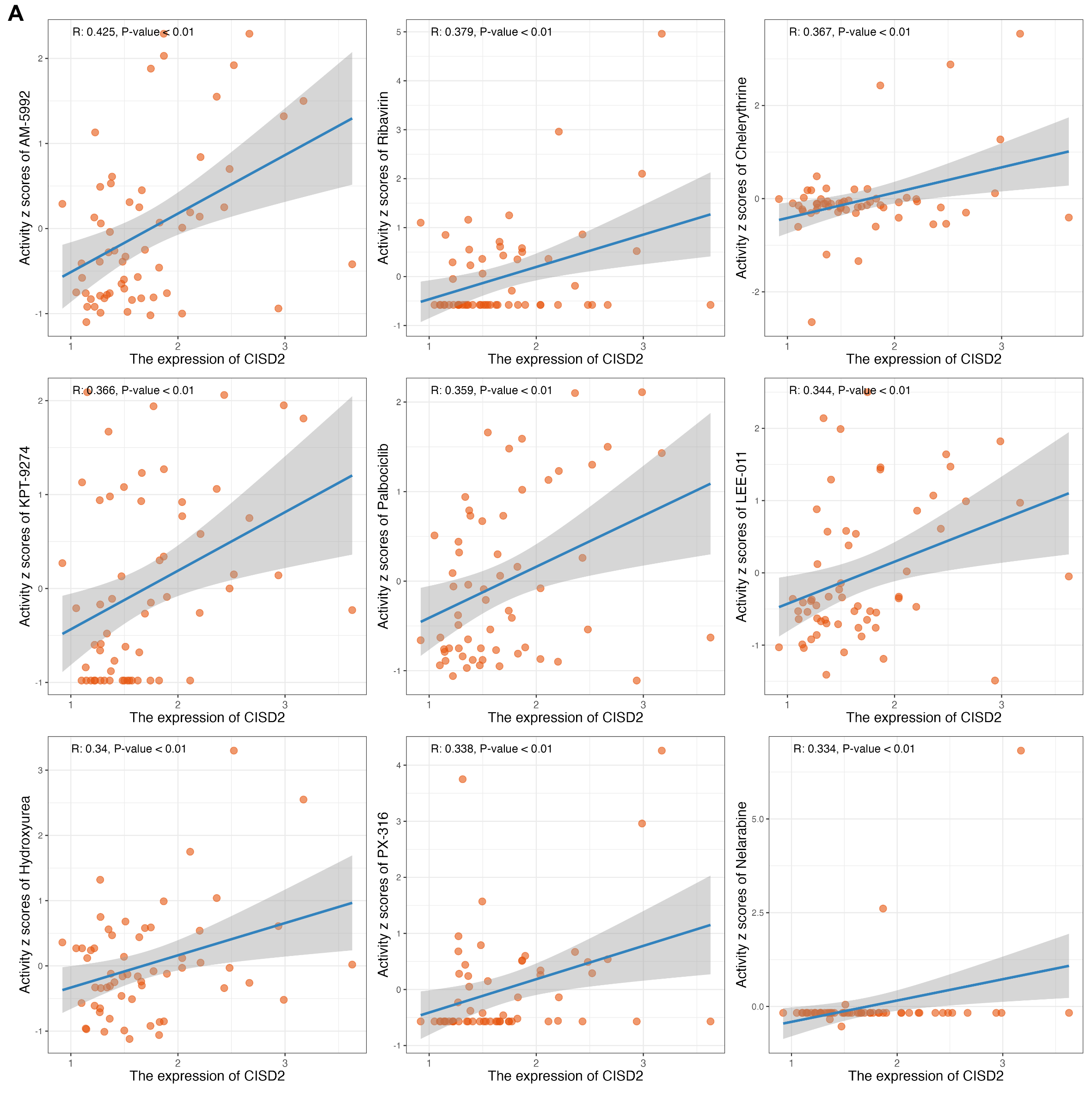


**Supplementary Figure S2.** The drug sensitivity assessment. **A.** The scatter plots included 9 drugs (AM-5992, Ribavirin, Chelerythrine, KPT-9274, Palbociclib, LEE-011, Hydroxyurea, PX-316, and Nelarabine) were drown by analyzed the correction of CISD2 expression and 9 drugs correlated positively.


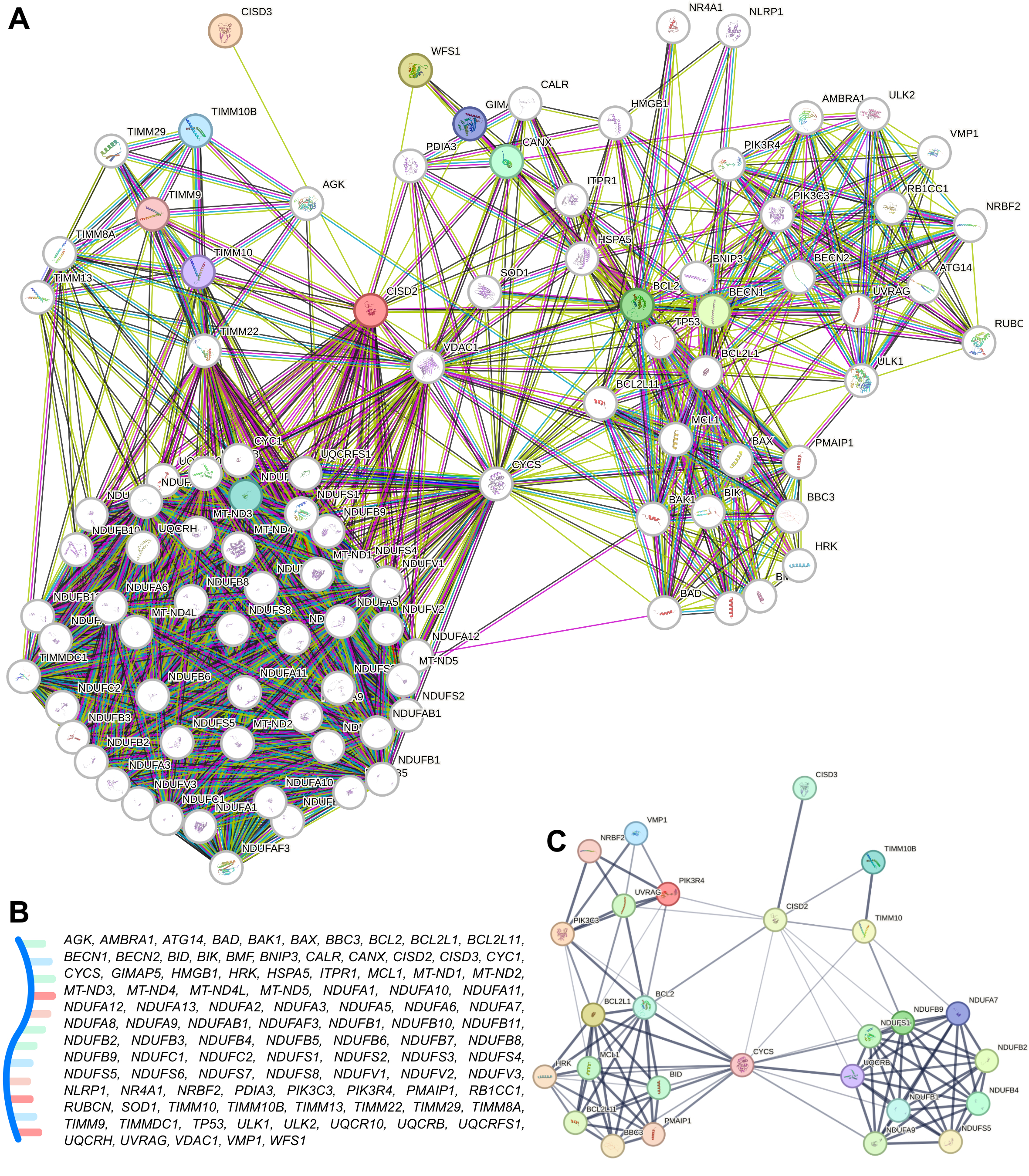
 **Supplementary Figure S3.** The CISD2 related genes and risk model (CISD2Risk). **A.** The PPI plot of 100 CISD2-related genes using STRING. **B.** The 100 genes CISD2-related genes. **C.** The PPI network of 27 genes enrolled in DLBCL patients was visualized by the STRING (version 12) online. DLBCL, Diffuse large B-cell lymphoma; PPI, protein-protein interaction; STRING, Search Tool for the Retrieval of Interacting Genes/Proteins.


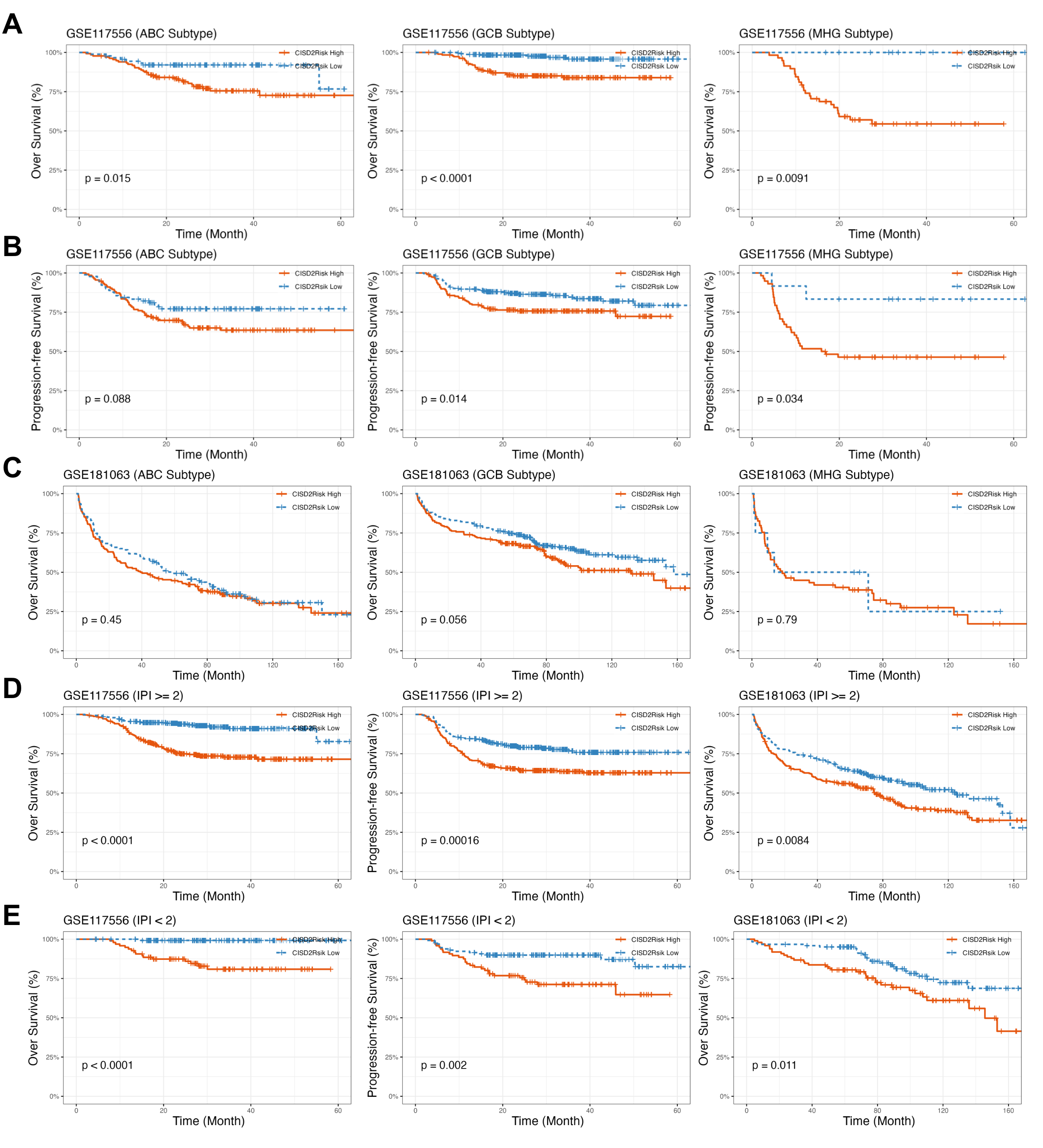


**Supplementary Figure S4.** Association between clinical features in DLBCL and CISD2Risk value that was divided into high- and low- groups according to the median based on GSE117556 (training dataset) and GSE181063 (validation dataset). **A.** The KM curves showed that high CISD2Risk group had poor OS among COO in the GSE117556. **B.** The KM curves showed that high CISD2Risk group had poor PFS among COO in the GSE117556. **C.** The KM curves showed that high CISD2Risk group had poor OS among COO in the GSE181063. **D.** The KM curves showed that high CISD2Risk group had poor prognosis (OS and PFS) in DLBCL patients with IPI >= 2. **E.** The KM curves showed that high CISD2Risk group had poor prognosis (OS and PFS) in DLBCL patients with IPI < 2. ABC, activated B-cell; CR, complete response; COO, cell of origin, DLBCL, Diffuse large B-cell lymphoma; GCB, germinal center B-cell; GSE, Gene Expression Omnibus Series; KM, Kaplan–Meier; MHG, molecular high-grade; IPI, international prognostic index; OS, over survival; PD, progressive disease; PFS, progression-free survival; PR, partial response; SD, stable disease.


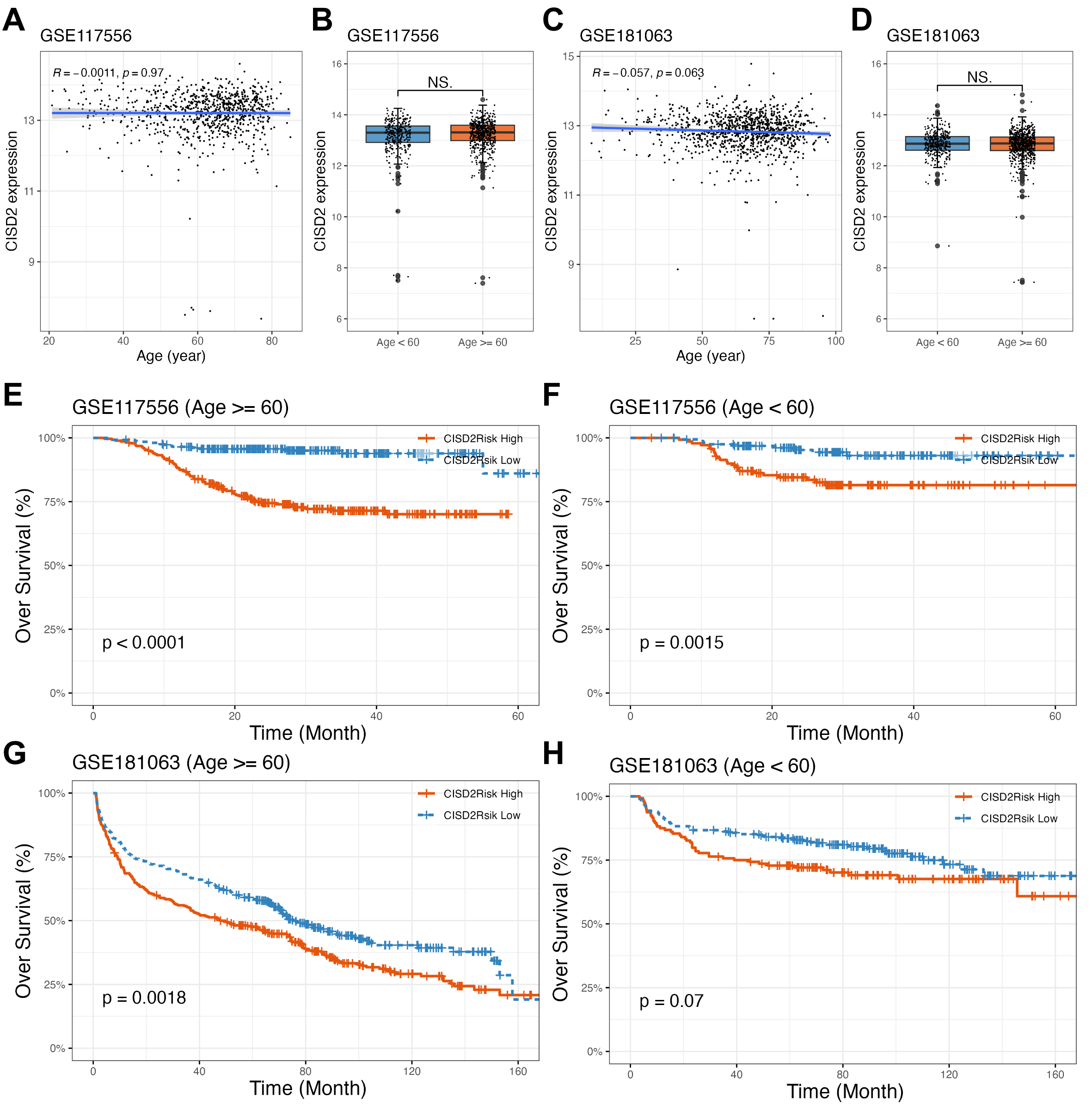


**Supplementary Figure S5.** The correction CISD2 expression with different age group. **A and C.** the scatter plots of linear regression of CISD2 expression and age both GSE117556 and GSE181063 datasets. **B and D.** the different expression analysis showed there was no significant between DLBCL patients with age >= 60 and age < 60. **E and F.** the KM plots showed that the poor OS was associated with high CISD2Risk value both DLBCL patients with age >= 60 and age < 60 in GSE117556 dataset. **G and H.** In GSE181063 dataset, the KM plots showed that the poor OS was associated with high CISD2Risk value in DLBCL patients with age >= 60, meanwhile, high CISD2Risk value was not indicated the significant difference in DLBCL patients with age < 60 (P = 0.07). GSE, Gene Expression Omnibus Series; KM, Kaplan–Meier; OS, over survival. ns, not signifcance.


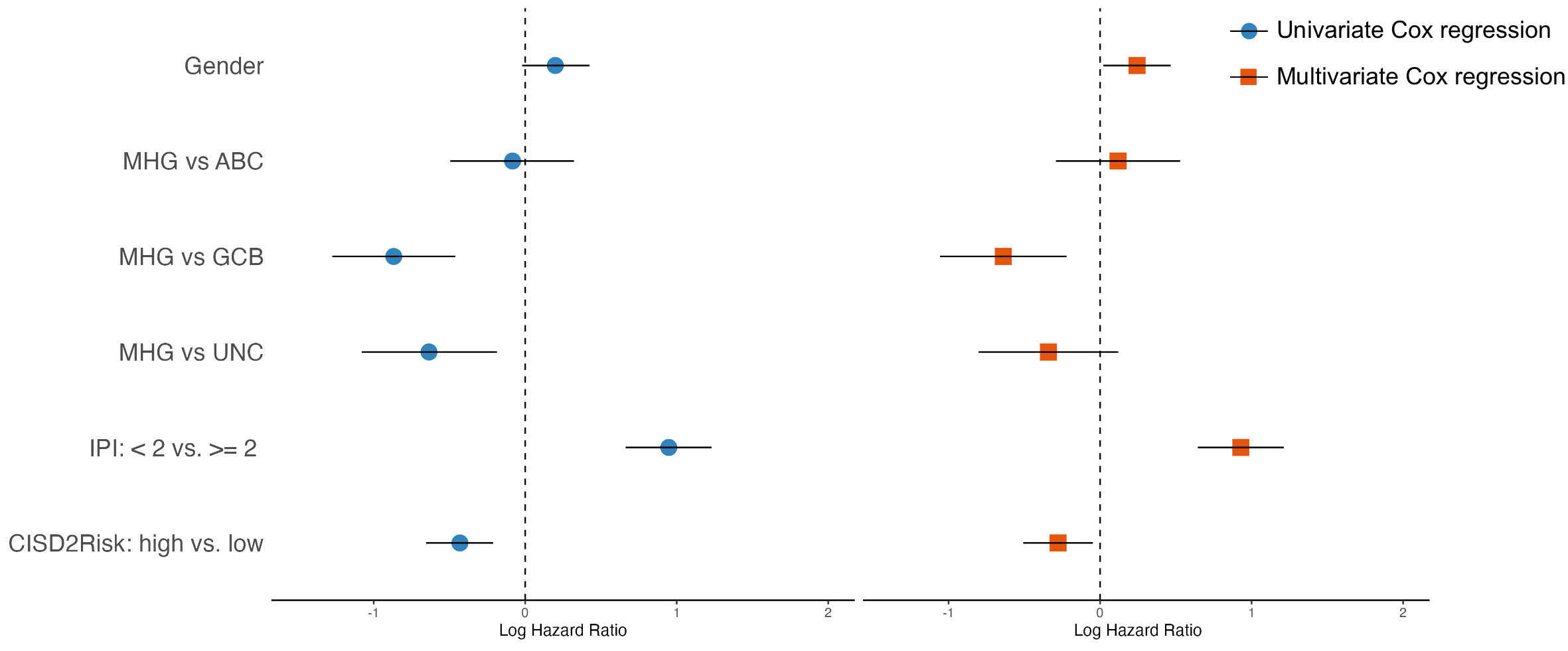


**Supplementary Figure S6.** The hazard ratios of clinical features integrated into the OS showed in the forest plots in DLBCL using univariate and multivariate cox regression analysis in the GSE181063 dataset, left, blue and square, univariate cox regression; right, red and circle;, multivariate cox regression; univariate Cox regression analysis; blue and square, multivariate Cox regression analysis. OS, over survival; PFS, progression-free survival.


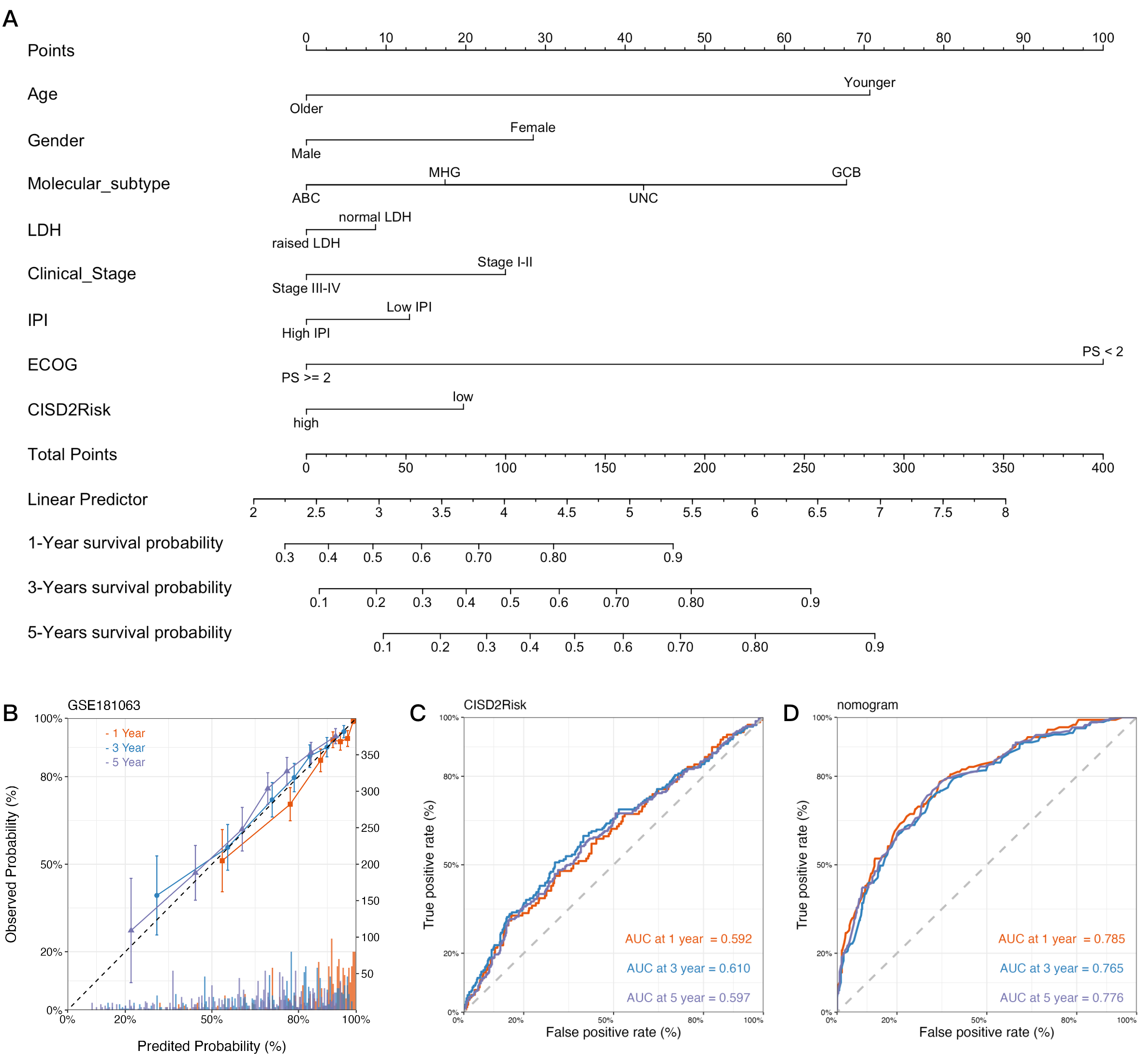


**Supplementary Figure S7.** The construction and validation of the nomogram. **A.** The nomogram plot of the GSE181063 dataset showed the prediction of clinical features including age, gender, molecular subgroup, LDH, clinical stage, IPI, ECOG PS, CISD2Risk, and 1-year, 3-year, and 5-year survival probability. **B.** The calibration curve of 1-year, 3-years, and 5-year survival probability of DLBCL patients, The dashed line represented a perfect uniformity between predicted probability and observed probability. The time-dependent ROC curves for nomogram (**C.**) and CISD2Risk (**D.**) at 1-year, 3-year, and 5-year for DLBCL, respectively. DLBCL, Diffuse large B-cell lymphoma; ECOG PS, Eastern Cooperative Oncology Group performance status; GSE, Gene Expression Omnibus Series; IPI, international prognostic index; LDH, lactate dehydrogenase; ROC, receiver operating characteristic.

## Supplementary Tables

Table S1. Basic data of public datasets enrolled in this study

| Datasets | Platform | Type | Samples(all) |
| --- | --- | --- | --- |
| TCGA-DLBC | Illumina | high throughput sequencing | 48 |
| GTEx | Illumina | gene expression RNA-sequencing | 7,845 |
| GSE83632 | GPL5175 | Expression profiling by array | 163 |
| GSE31312 | GPL570 | Expression profiling by array | 498 |
| GSE32918 | GPL8432 | Expression profiling by array | 172 |
| GSE93984 | GPL570 | Expression profiling by array | 88 |
| GSE117556 | GPL14951 | Expression profiling by array | 928 |
| GSE181063 | GPL14951 | Expression profiling by array | 1311 |

Table S2. Primer of sequences

| Gene symbol | Primer (5’ - 3’) |
| --- | --- |
| CISD2 | F: GTGGCCCGTATCGTGAAGG |
|  | R: CTAGCGAACCCGGTAATGCTT |
| β-actin | F: TGACGTGGACATCCGCAAAG |
|  | R: CTGGAAGGTGGACAGCGAGG |

Table S3. Drug Sensitivity Assessment

| Drug | Pearson | Pval |
| --- | --- | --- |
| AM-5992 | 0.4250387 | 7.919869e-04 |
| Ribavirin | 0.3785252 | 3.115066e-03 |
| Chelerythrine | 0.3672481 | 4.219782e-03 |
| KPT-9274 | 0.3662350 | 4.334190e-03 |
| Palbociclib | 0.3588507 | 5.254112e-03 |
| LEE-011 | 0.3438852 | 7.658123e-03 |
| Hydroxyurea | 0.3398422 | 8.453280e-03 |
| PX-316 | 0.3383863 | 8.756730e-03 |
| Nelarabine | 0.3335347 | 9.837335e-03 |
| ZSTK-474 | -0.3438784 | 7.659404e-03 |
| Apitolisib | -0.3442773 | 7.584595e-03 |
| CC-223 | -0.3460249 | 7.264326e-03 |
| GSK-2126458 | -0.3464201 | 7.193554e-03 |
| KU-55933 | -0.3513686 | 6.357132e-03 |
| VS-5584 | -0.3522676 | 6.214680e-03 |
| P-529 | -0.3561636 | 5.629170e-03 |
| Pp-242 | -0.3590501 | 5.227179e-03 |
| Pluripotin | -0.3625615 | 4.772354e-03 |
| Deforolimius | -0.3960819 | 1.900596e-03 |
| GDC-0349 | -0.4024086 | 1.580091e-03 |
| Everolimus | -0.4070344 | 1.377352e-03 |
| AZD-3147 | -0.4180506 | 9.853077e-04 |
| PQR-620 | -0.4279116 | 7.229858e-04 |
| AZD-8055 | -0.4340692 | 5.930583e-04 |
| INK-128 | -0.4436879 | 4.319410e-04 |
| LY-3023414 | -0.5085224 | 3.920164e-05 |

Table S4. GO enrichment analysis

| Category | Term | Count | % | PValue | Pop Hits | Fold Enrichment | Bonferroni | Benjamini | FDR |
| --- | --- | --- | --- | --- | --- | --- | --- | --- | --- |
| BP | GO:0009060~aerobic respiration | 9 | 36.0 | 6.92E-15 | 68 | 107.06 | 2.06E-12 | 2.07E-12 | 1.793E-12 |
| BP | GO:0006120~mitochondrial electron transport, NADH to ubiquinone | 8 | 32.0 | 7.27E-14 | 47 | 137.68 | 2.18E-11 | 1.09E-11 | 9.4E-12 |
| BP | GO:0042776~mitochondrial ATP synthesis coupled proton transport | 8 | 32.0 | 7.94E-13 | 65 | 99.55 | 2.38E-10 | 7.94E-11 | 6.8E-11 |
| BP | GO:0001836~release of cytochrome c from mitochondria | 6 | 24.0 | 4.86E-11 | 23 | 211.02 | 1.458E-8 | 3.645E-9 | 3.147E-9 |
| BP | GO:0032981~mitochondrial respiratory chain complex I assembly | 7 | 28.00 | 9.75E-11 | 64 | 88.47 | 2.92E-8 | 5.850E-9 | 5.05E-9 |
| BP | GO:0090200~positive regulation of release of cytochrome c from mitochondria | 5 | 20.0 | 2.57E-8 | 27 | 149.79 | 7.7E-6 | 1.289E-6 | 1.11E-6 |
| BP | GO:0006915~apoptotic process | 9 | 36.0 | 3.15E-7 | 616 | 11.81 | 9.47E-5 | 1.353E-5 | 1.168E-5 |
| BP | GO:0043065~positive regulation of apoptotic process | 7 | 28.00 | 1.728E-6 | 327 | 17.31 | 5.18E-4 | 6.48E-5 | 5.59E-5 |
| BP | GO:0097192~extrinsic apoptotic signaling pathway in absence of ligand | 4 | 16.0 | 9.27E-6 | 35 | 92.44 | 0.0027 | 3.091E-4 | 2.668E-4 |
| BP | GO:2001244~positive regulation of intrinsic apoptotic signaling pathway | 4 | 16.0 | 1.099E-5 | 37 | 87.45 | 0.0032 | 3.297E-4 | 2.847E-4 |
| BP | GO:0032465~regulation of cytokinesis | 4 | 16.0 | 1.617E-5 | 42 | 77.03 | 0.0048 | 4.41E-4 | 3.809E-4 |
| BP | GO:0008630~intrinsic apoptotic signaling pathway in response to DNA damage | 4 | 16.0 | 2.914E-5 | 51 | 63.44 | 0.0087 | 7.28E-4 | 6.29E-4 |
| BP | GO:0031334~positive regulation of protein complex assembly | 4 | 16.0 | 3.46E-5 | 54 | 59.91 | 0.0103 | 7.99E-4 | 6.90E-4 |
| BP | GO:0042149~cellular response to glucose starvation | 4 | 16.0 | 4.29E-5 | 58 | 55.78 | 0.0128 | 9.20E-4 | 7.9E-4 |
| BP | GO:1902237~positive regulation of ER stress-induced intrinsic apoptotic signaling pathway | 3 | 12.0 | 7.33E-5 | 11 | 220.61 | 0.0217 | 0.0014 | 0.0012 |
| BP | GO:0046902~regulation of mitochondrial membrane permeability | 3 | 12.0 | 8.79E-5 | 12 | 202.22 | 0.0260 | 0.0016 | 0.0014 |
| BP | GO:0010506~regulation of autophagy | 4 | 16.0 | 9.65E-5 | 76 | 42.57 | 0.0285 | 0.0017 | 0.0014 |
| BP | GO:0006919~activatie-type endopeptidase activity involved in apoptotic process | 4 | 16.0 | 1.16E-4 | 81 | 39.94 | 0.0343 | 0.0019 | 0.0016 |
| BP | GO:0080135~regulation of cellular response to stress | 3 | 12.0 | 1.21E-4 | 14 | 173.33 | 0.0356 | 0.0019 | 0.0016 |
| BP | GO:0008637~apoptotic mitochondrial changes | 3 | 12.0 | 1.806E-4 | 17 | 142.75 | 0.0527 | 0.0027 | 0.0023 |
| BP | GO:2000811~negative regulation of anoikis | 3 | 12.0 | 2.26E-4 | 19 | 127.72 | 0.0657 | 0.0032 | 0.0027 |
| BP | GO:0036092~phosphatidylinositol-3-phosphate biosynthetic process | 3 | 12.0 | 2.78E-4 | 21 | 115.55 | 0.0800 | 0.0037 | 0.0032 |
| BP | GO:2001243~negative regulation of intrinsic apoptotic signaling pathway | 3 | 12.0 | 4.98E-4 | 28 | 86.669 | 0.1388 | 0.0064 | 0.0056 |
| BP | GO:0051881~regulation of mitochondrial membrane potential | 3 | 12.0 | 6.51E-4 | 32 | 75.835 | 0.1776 | 0.0081 | 0.0070 |
| BP | GO:2001240~negative regulation of extrinsic apoptotic signaling pathway in absence of ligand | 3 | 12.0 | 8.71E-4 | 37 | 65.587 | 0.2302 | 0.0100 | 0.0086 |
| BP | GO:0043029~T cell homeostasis | 3 | 12.0 | 8.71E-4 | 37 | 65.587 | 0.2302 | 0.0100 | 0.0086 |
| BP | GO:0072593~reactive oxygen species metabolic process | 3 | 12.0 | 9.19E-4 | 38 | 63.861 | 0.2412 | 0.0102 | 0.0088 |
| BP | GO:0045333~cellular respiration | 3 | 12.0 | 0.0011 | 42 | 57.77 | 0.2861 | 0.0120 | 0.0103 |
| BP | GO:0097352~autophagosome maturation | 3 | 12.0 | 0.0011 | 43 | 56.43 | 0.2976 | 0.0121 | 0.0105 |
| BP | GO:0034097~response to cytokine | 3 | 12.0 | 0.0014 | 48 | 50.55 | 0.3558 | 0.0146 | 0.0126 |
| BP | GO:0043280~positive regulatie-type endopeptidase activity involved in apoptotic process | 3 | 12.0 | 0.0016 | 51 | 47.58 | 0.3911 | 0.0159 | 0.01380 |
| BP | GO:0010507~negative regulation of autophagy | 3 | 12.0 | 0.0022 | 59 | 41.13 | 0.4842 | 0.0206 | 0.01784 |
| BP | GO:0097048~dendritic cell apoptotic process | 2 | 8.0 | 0.0023 | 2 | 808.91 | 0.5089 | 0.02152 | 0.0185 |
| BP | GO:0051402~neuron apoptotic process | 3 | 12.0 | 0.0038 | 78 | 31.11 | 0.6825 | 0.0336 | 0.0290 |
| BP | GO:0034976~response to endoplasmic reticulum stress | 3 | 12.0 | 0.0044 | 84 | 28.88 | 0.7345 | 0.0378 | 0.0326 |
| BP | GO:0006974~cellular response to DNA damage stimulus | 4 | 16.0 | 0.0047 | 292 | 11.08 | 0.7619 | 0.0397 | 0.0343 |
| BP | GO:0030242~pexophagy | 2 | 8.0 | 0.0059 | 5 | 323.56 | 0.8310 | 0.0479 | 0.0413 |
| BP | GO:0008584~male gonad development | 3 | 12.0 | 0.0073 | 109 | 22.263 | 0.8895 | 0.0577 | 0.0498 |
| BP | GO:0042773~ATP synthesis coupled electron transport | 2 | 8.0 | 0.0082 | 7 | 231.11 | 0.9170 | 0.0635 | 0.0548 |
| BP | GO:1903896~positive regulation of IRE1-mediated unfolded protein response | 2 | 8.0 | 0.0094 | 8 | 202.22 | 0.9418 | 0.0708 | 0.0611 |
| BP | GO:2000669~negative regulation of dendritic cell apoptotic process | 2 | 8.0 | 0.0106 | 9 | 179.75 | 0.9592 | 0.0776 | 0.0670 |
| BP | GO:0071456~cellular response to hypoxia | 3 | 12.0 | 0.0108 | 134 | 18.11 | 0.9624 | 0.0777 | 0.0670 |
| BP | GO:2000271~positive regulation of fibroblast apoptotic process | 2 | 8.0 | 0.0117 | 10 | 161.78 | 0.9714 | 0.0803 | 0.0693 |
| BP | GO:0043583~ear development | 2 | 8.0 | 0.0117 | 10 | 161.78 | 0.9714 | 0.0803 | 0.0693 |
| BP | GO:0006914~autophagy | 3 | 12.0 | 0.0143 | 155 | 15.65 | 0.9869 | 0.0957 | 0.0826 |
| BP | GO:0032801~receptor catabolic process | 2 | 8.0 | 0.0152 | 13 | 124.4 | 0.9901 | 0.0997 | 0.0861 |
| BP | GO:0097284~hepatocyte apoptotic process | 2 | 8.0 | 0.0164 | 14 | 115.55 | 0.9931 | 0.1029 | 0.0888 |
| BP | GO:0006122~mitochondrial electron transport, ubiquinol to cytochrome c | 2 | 8.0 | 0.0164 | 14 | 115.55 | 0.9931 | 0.1029 | 0.0888 |
| BP | GO:1902230~negative regulation of intrinsic apoptotic signaling pathway in response to DNA damage | 2 | 8.0 | 0.0176 | 15 | 107.8 | 0.9951 | 0.1079 | 0.0931 |
| BP | GO:0001782~B cell homeostasis | 2 | 8.0 | 0.0303 | 26 | 62.22 | 0.9999 | 0.1786 | 0.1542 |
| BP | GO:0045022~early endosome to late endosome transport | 2 | 8.0 | 0.0303 | 26 | 62.22 | 0.9999 | 0.1786 | 0.1542 |
| BP | GO:0051607~defense response to virus | 3 | 12.0 | 0.0309 | 234 | 10.37 | 0.9999 | 0.1787 | 0.1543 |
| BP | GO:0006622~protein targeting to lysosome | 2 | 8.0 | 0.0326 | 28 | 57.77 | 0.9999 | 0.1845 | 0.1592 |
| BP | GO:0042981~regulation of apoptotic process | 3 | 12.0 | 0.0332 | 243 | 9.986 | 0.9999 | 0.1845 | 0.1592 |
| BP | GO:0006626~protein targeting to mitochondrion | 2 | 8.0 | 0.0349 | 30 | 53.92 | 0.9999 | 0.1907 | 0.1646 |
| BP | GO:0070059~intrinsic apoptotic signaling pathway in response to endoplasmic reticulum stress | 2 | 8.0 | 0.0418 | 36 | 44.93 | 0.9999 | 0.2200 | 0.1899 |
| BP | GO:0097193~intrinsic apoptotic signaling pathway | 2 | 8.0 | 0.0418 | 36 | 44.93 | 0.9999 | 0.2200 | 0.1899 |
| BP | GO:0048536~spleen development | 2 | 8.0 | 0.0429 | 37 | 43.72 | 0.9999 | 0.2221 | 0.1918 |
| BP | GO:0008625~extrinsic apoptotic signaling pathway via death domain receptors | 2 | 8.0 | 0.0463 | 40 | 40.44 | 0.9999 | 0.2357 | 0.2034 |
| BP | GO:0001541~ovarian follicle development | 2 | 8.0 | 0.0474 | 41 | 39.45 | 0.9999 | 0.2374 | 0.2049 |
| BP | GO:0048538~thymus development | 2 | 8.0 | 0.0542 | 47 | 34.42 | 0.9999 | 0.2668 | 0.2303 |
| BP | GO:0016241~regulation of macroautophagy | 2 | 8.0 | 0.0632 | 55 | 29.41 | 0.9999 | 0.3058 | 0.2640 |
| BP | GO:0043525~positive regulation of neuron apoptotic process | 2 | 8.0 | 0.0654 | 57 | 28.38 | 0.9999 | 0.3115 | 0.2689 |
| BP | GO:0016236~macroautophagy | 2 | 8.0 | 0.0786 | 69 | 23.44 | 0.9999 | 0.3687 | 0.3183 |
| CC | GO:0005739~mitochondrion | 18 | 72.0 | 5.36E-15 | 1458 | 10.18 | 3.41E-13 | 3.43E-13 | 2.95E-13 |
| CC | GO:0005747~mitochondrial respiratory chain complex I | 8 | 32.0 | 1.23E-13 | 51 | 129.40 | 7.89E-12 | 3.94E-12 | 3.39E-12 |
| CC | GO:0005743~mitochondrial inner membrane | 12 | 48.0 | 1.96E-12 | 483 | 20.495 | 1.260E-10 | 4.2E-11 | 3.60E-11 |
| CC | GO:0005741~mitochondrial outer membrane | 8 | 32.0 | 2.68E-9 | 206 | 32.037 | 1.71E-7 | 4.29E-8 | 3.69E-8 |
| CC | GO:0035032~phosphatidylinositol 3-kinase complex, class III | 4 | 16.0 | 2.76E-8 | 6 | 549.97 | 1.76E-6 | 3.53E-7 | 3.0E-7 |
| CC | GO:0097136~Bcl-2 family protein complex | 4 | 16.0 | 7.72E-8 | 8 | 412.48 | 4.94E-6 | 8.23E-7 | 7.08E-7 |
| CC | GO:0005758~mitochondrial intermembrane space | 4 | 16.0 | 1.42E-4 | 88 | 37.49 | 0.0090 | 0.0013 | 0.0011 |
| CC | GO:0070469~respiratory chain | 3 | 12.0 | 4.475E-4 | 27 | 91.66 | 0.0282 | 0.0035 | 0.0030 |
| CC | GO:0097143~PUMA-BCL-xl complex | 2 | 8.0 | 0.0023 | 2 | 824.96 | 0.1384 | 0.0165 | 0.0142 |
| CC | GO:0034271~phosphatidylinositol 3-kinase complex, class III, type I | 2 | 8.0 | 0.0046 | 4 | 412.48 | 0.2577 | 0.0270 | 0.0232 |
| CC | GO:0034272~phosphatidylinositol 3-kinase complex, class III, type II | 2 | 8.0 | 0.0046 | 4 | 412.48 | 0.2577 | 0.0270 | 0.0232 |
| CC | GO:0005770~late endosome | 3 | 12.0 | 0.0137 | 154 | 16.07 | 0.5871 | 0.0732 | 0.0629 |
| CC | GO:0005783~endoplasmic reticulum | 5 | 20.0 | 0.0426 | 1157 | 3.56 | 0.9387 | 0.2102 | 0.1806 |
| CC | GO:0005759~mitochondrial matrix | 3 | 12.0 | 0.0755 | 392 | 6.31 | 0.9934 | 0.3455 | 0.2969 |
| CC | GO:0030670~phagocytic vesicle membrane | 2 | 8.0 | 0.0859 | 77 | 21.42 | 0.9968 | 0.3478 | 0.2989 |
| CC | GO:0005776~autophagosome | 2 | 8.0 | 0.0869 | 78 | 21.15 | 0.9970 | 0.3478 | 0.2989 |
| MF | GO:0008137~NADH dehydrogenase (ubiquinone) activity | 8 | 32.0 | 7.42E-14 | 44 | 137.78 | 3.04E-12 | 3.04E-12 | 2.895E-12 |
| MF | GO:0051434~BH3 domain binding | 3 | 12.0 | 2.29E-5 | 6 | 378.9 | 9.42E-4 | 4.71E-4 | 4.48E-4 |
| MF | GO:0051537~2 iron, 2 sulfur cluster binding | 3 | 12.0 | 5.69E-4 | 28 | 81.19 | 0.0230 | 0.0077 | 0.0074 |
| MF | GO:0042803~protein homodimerization activity | 5 | 20.0 | 0.0129 | 736 | 5.14 | 0.4132 | 0.1324 | 0.1259 |
| MF | GO:0015267~channel activity | 2 | 8.0 | 0.0324 | 26 | 58.29 | 0.7413 | 0.2660 | 0.2530 |
| MF | GO:0005515~protein binding | 21 | 84.0 | 0.0603 | 12648 | 1.25 | 0.9221 | 0.4125 | 0.3923 |
| MF | GO:0046982~protein heterodimerization activity | 3 | 12.0 | 0.0806 | 374 | 6.07 | 0.9682 | 0.4179 | 0.3976 |
| MF | GO:0009055~electron carrier activity | 2 | 8.0 | 0.0815 | 67 | 22.62 | 0.9694 | 0.4179 | 0.3976 |

Table S5. KEGG pathway analysis

| Term | Count | % | PValue | Pop Hits | Fold Enrichment | Bonferroni |
| --- | --- | --- | --- | --- | --- | --- |
| hsa05014:Amyotrophic lateral sclerosis | 16 | 64.0 | 1.01E-16 | 364 | 16.91 | 8.7E-15 |
| hsa05022:Pathways of neurodegeneration - multiple diseases | 16 | 64.0 | 5.6E-15 | 476 | 12.93 | 4.47E-13 |
| hsa04932:Non-alcoholic fatty liver disease | 12 | 48.0 | 1.63E-14 | 155 | 29.78 | 1.28E-12 |
| hsa05016:Huntington disease | 14 | 56.0 | 2.1E-14 | 306 | 17.60 | 1.73E-12 |
| hsa05010:Alzheimer disease | 14 | 56.0 | 4.13E-13 | 384 | 14.02 | 3.E-11 |
| hsa00190:Oxidative phosphorylation | 10 | 40.0 | 1.19E-11 | 134 | 28.71 | 9.4E-10 |
| hsa04215:Apoptosis - multiple species | 7 | 28.0 | 9.2E-11 | 32 | 84.16 | 7.3E-9 |
| hsa05012:Parkinson disease | 11 | 44.0 | 2.06E-10 | 266 | 15.91 | 1.63E-8 |
| hsa04210:Apoptosis | 9 | 36.0 | 6.1E-10 | 136 | 25.46 | 4.8E-8 |
| hsa05020:Prion disease | 10 | 40.0 | 6.9E-9 | 273 | 14.09 | 5.E-7 |
| hsa05415:Diabetic cardiomyopathy | 9 | 36.0 | 1.48E-8 | 203 | 17.05 | 1.17E-6 |
| hsa05208:Chemical carcinogenesis - reactive oxygen species | 9 | 36.0 | 3.09E-8 | 223 | 15.52 | 2.4E-6 |
| hsa04723:Retrograde endocannabinoid signaling | 8 | 32.0 | 4.1E-8 | 148 | 20.79 | 3.2E-6 |
| hsa04714:Thermogenesis | 9 | 36.0 | 4.2E-8 | 232 | 14.92 | 3.3E-6 |
| hsa01524:Platinum drug resistance | 6 | 24.0 | 7.5E-7 | 73 | 31.62 | 5.9E-5 |
| hsa04115:p53 signaling pathway | 6 | 24.0 | 8.1E-7 | 74 | 31.19 | 6.4E-5 |
| hsa04140:Autophagy - animal | 6 | 24.0 | 1.96E-5 | 141 | 16.37 | 0.0015 |
| hsa05210:Colorectal cancer | 5 | 20.0 | 5.2E-5 | 86 | 22.37 | 0.0041 |
| hsa05162:Measles | 5 | 20.0 | 3.35E-4 | 139 | 13.84 | 0.0261 |
| hsa05200:Pathways in cancer | 7 | 28.0 | 0.0014 | 531 | 5.07 | 0.1067 |
| hsa01100:Metabolic pathways | 11 | 44.0 | 0.0019 | 1541 | 2.74 | 0.1421 |
| hsa05131:Shigellosis | 5 | 20.0 | 0.0028 | 247 | 7.78 | 0.2026 |
| hsa05017:Spinocerebellar ataxia | 4 | 16.0 | 0.0050 | 143 | 10.76 | 0.3282 |
| hsa05152:Tuberculosis | 4 | 16.0 | 0.0094 | 180 | 8.55 | 0.5290 |
| hsa05169:Epstein-Barr virus infection | 4 | 16.0 | 0.0129 | 202 | 7.61 | 0.6434 |
| hsa05170:Human immunodeficiency virus 1 infection | 4 | 16.0 | 0.0147 | 212 | 7.25 | 0.6912 |
| hsa05417:Lipid and atherosclerosis | 4 | 16.0 | 0.0153 | 215 | 7.15 | 0.7049 |
| hsa01521:EGFR tyrosine kinase inhibitor resistance | 3 | 12.0 | 0.0161 | 79 | 14.61 | 0.7226 |
| hsa05222:Small cell lung cancer | 3 | 12.0 | 0.0214 | 92 | 12.54 | 0.8197 |
| hsa05145:Toxoplasmosis | 3 | 12.0 | 0.0309 | 112 | 10.30 | 0.9164 |
| hsa04151:PI3K-Akt signaling pathway | 4 | 16.0 | 0.0551 | 354 | 4.34 | 0.9887 |
| hsa05161:Hepatitis B | 3 | 12.0 | 0.0602 | 162 | 7.12 | 0.9926 |
| hsa04630:JAK-STAT signaling pathway | 3 | 12.0 | 0.0629 | 166 | 6.95 | 0.9941 |
| hsa04136:Autophagy - other | 2 | 8.0 | 0.0765 | 32 | 24.04 | 0.9981 |
| hsa05167:Kaposi sarcoma-associated herpesvirus infection | 3 | 12.0 | 0.08255 | 194 | 5.95 | 0.9988 |

Table S6. AUC of time-dependent ROC curves

| Dataset | Type | Time | Hazard Ratio | 95% confidence interval |
| --- | --- | --- | --- | --- |
| GSE117556 | nomogram | 1 year | 72.87 | 66.58-79.16 |
|  |  | 3 year | 75.54 | 70.15-80.93 |
|  |  | 5 year | 81.76 | 65.65-97.87 |
|  | CISD2Risk | 1 year | 74.68 | 67.53-81.83 |
|  |  | 3 year | 77.29 | 72.00-82.58 |
|  |  | 5 year | 86.40 | 74.64-98.16 |
| GSE181063 | nomogram | 1 year | 78.49 | 74.04-82.94 |
|  |  | 3 year | 76.48 | 72.56-80.40 |
|  |  | 5 year | 77.64 | 74.01-81.27 |
|  | CISD2Risk | 1 year | 59.19 | 53.47-64.91 |
|  |  | 3 year | 61.04 | 56.22-65.86 |
|  |  | 5 year | 59.74 | 55.21-64.27 |
